# Colossal magnetic tunability of excitons in 2D van der Waals magnetic semiconductor CrSBr

**DOI:** 10.1038/s41467-026-77986-z

**Published:** 2026-09-24

**Authors:** Rafał Komar, Mateusz Goryca, Aleksandra Łopion, Miłosz Rybak, Tomasz Woźniak, Mateusz Raczyński, Karol M. Gała̧zka, Kseniia Mosina, Aljoscha Söll, Zdeněk Sofer, Wojciech Pacuski, Clément Faugeras, Magdalena Birowska, Piotr Kossacki, Tomasz Kazimierczuk

**Affiliations:** 1 Institute of Experimental Physics, Faculty of Physics, University of Warsaw, Warsaw, Poland; 2 Institute of Physics, University of Muenster, Muenster, Germany; 3 Faculty of Fundamental Problems of Technology, Wrocław University of Science and Technology, Wrocław, Poland; 4 Institute of Theoretical Physics, Faculty of Physics, University of Warsaw, Warsaw, Poland; 5 Department of Inorganic Chemistry, University of Chemistry and Technology Prague, Prague, Czech Republic; 6 LNCMI, UPR 3228, CNRS, EMFL, Université Grenoble Alpes, Grenoble, France

**Keywords:** Two-dimensional materials, Magnetic properties and materials

## Abstract

2D magnetic semiconductors, which intrinsically couple a rich landscape of magnetic orders with tightly bound electron-hole pairs (excitons), present an exciting platform to investigate the interplay between optical and magnetic phenomena at the atomic scale. In such systems, the strength of magneto-optical effects determines how deeply the magnetic properties can be revealed. Here, we report the observation of remarkably strong magneto-optical effects in the 2D magnetic semiconductor CrSBr that allow probing its magnetic order with unprecedented sensitivity. By investigating optical transitions above the fundamental exciton energy, we discover massive spectral red- and blueshifts approaching 100 meV under applied magnetic fields – an order of magnitude larger than previously observed magneto-excitonic responses. Our comprehensive magneto-optical experiments, accompanied by detailed DFT calculations, indicate the possible origin of the transitions exhibiting such intriguing behavior. These findings open avenues for exploiting magneto-optical phenomena in newly accessible regimes, enabling novel opto-spintronic applications previously limited by weak magnetic responses.

## Abstract (web summary)

CrSBr is a van der Waals magnetic semiconductor with an exciton energy strongly influenced by the magnetic ordering of the material. Here, focussing on the spectral range above that of the fundamental excitonic transitions, Komar and coauthors find large energy shifts upon changes in magnetic ordering, of nearly 100 meV.

## Introduction

The ability to control the spin degree of freedom of charge carriers is a central pursuit in the rapidly evolving field of spintronics. The coupling between band carriers (electrons or holes) and localized magnetic moments in magnetic semiconductors provides a powerful means to achieve such control, giving rise to intriguing phenomena, such as the giant Zeeman effect and spin-polarized carrier transport. Among the plethora of material platforms harnessing this coupling, the recently emerged two-dimensional (2D) magnetic semiconductors^1–4^, wherein magnetic ions are incorporated into an atomically thin semiconductor host, have ushered in a new frontier of opportunities. These 2D systems offer a unique opportunity to explore the interplay between charge, spin, and magnetism at the ultimate scaling limit. One of the most captivating aspects of 2D magnetic semiconductors is the potential for exceptionally strong coupling between the band carriers and the localized d-electrons of the magnetic ions, facilitated by the reduced dimensionality and enhanced exchange interactions.

The ability to detect and characterize magnetic order in these 2D systems is crucial for understanding the underlying physics and developing spintronic applications. It is, however, experimentally challenging, fueling the development of specialized experimental techniques, such as NV-center magnetometry. Optical probes, often preferred due to the possibility of seamless integration with more complex experimental platforms, unfortunately lack universal signatures of magnetic order. Depending on the material, the magnetic properties may manifest as a rotation of the polarization in the magneto-optical Kerr effect^3^, a shift in the Raman-active magnon modes^5^, or a modulation of the polarization degree of the photoluminescence^6^.

In this respect, the air-stable^7^ chromium sulfide bromide (CrSBr) stands out due to particularly clear effects of the magnetic order on the optical spectrum^8^. In contrast to, e.g., earlier studied chromium trihalides, the excitons in CrSBr are more of Wannier character^8^ and therefore more easily affected by the magnetic order of the sample. The magnetic field dependence is strong enough that it allows for optically observing magnon excitations (spin waves) in the material in time-resolved experiments^9,10^.

In this work, we report on the observation of unprecedentedly strong magneto-optical effects in CrSBr, surpassing the response exhibited by any previously studied 2D semiconductor system. In contrast to prior studies, we focus on the less explored spectral range above the energy of fundamental excitonic transitions. We show that transitions in this energy range exhibit significantly stronger sensitivity to the magnetic order, manifesting as a colossal spectral change—both red- and blueshifts—approaching 100 meV, as evidenced by our comprehensive magneto-optical experiments with the magnetic field oriented along all three crystallographic directions. These experimental findings, substantiated by state-of-the-art density functional theory (DFT) calculations, enable us to discuss the possible origins of the observed extraordinary field-dependent behavior. Although our theoretical model does not yet provide a complete picture, it demonstrates that the magnitude and direction of the observed colossal spectral shifts are best understood as a direct consequence of the field-driven reconstruction of the underlying single-particle band structure. We hope these new results will stimulate further discussion and inspire more refined theoretical work, while charting a path toward harnessing magneto-optical phenomena with an unprecedented level of sensitivity – enabling novel experiments and applications previously constrained by the limited magneto-optical response of conventional systems.

## Results and discussion

### Basic properties

As a direct-gap van der Waals semiconductor, CrSBr exhibits prominent excitonic resonances visible both in reflectivity and in photoluminescence spectra. The orthorhombic crystalline symmetry^11^ results in strong anisotropy of direct band-gap excitations in the near-infrared energy range^8,9^. Showcasing the intriguing coupling of electronic and magnetic properties, the energy of these excitonic resonances is notably influenced by the magnetic ordering between the layers^8^. Below the critical temperature of 132 K, CrSBr is an A-type antiferromagnet^7,11–13^, wherein the spin orientation alternates from layer to layer, with consistent ferromagnetic (FM) intralayer ordering. With that, CrSBr offers three magnetic phase transitions^7,11,14^.

**Fig. 1:**
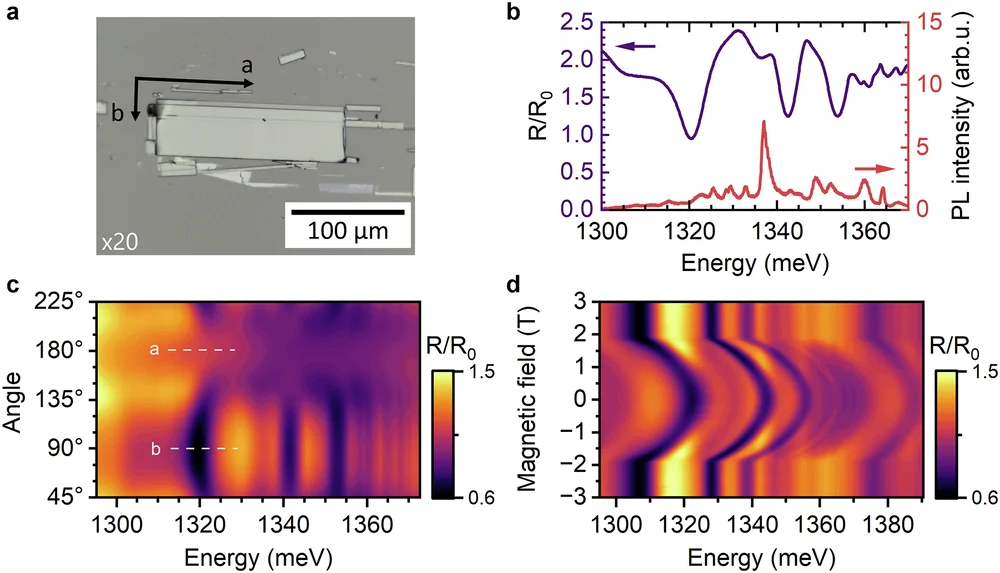
Basic properties of the CrSBr sample. **a** Optical microscope snapshot of a bulk CrSBr flake with crystallographic in-plane axes marked. The thickness of this flake was checked with the use of atomic force microscopy to be around 700 nm. **b** Reflectance and photoluminescence of a chosen bulk flake. **c** False-color plot of reflectance as a function of photon energy (horizontal) and light polarization angle with respect to the crystal *a*-axis (vertical). **d** Magnetoreflectance false-color plot of fundamental excitonic states with the magnetic field applied in the Faraday configuration (magnetic field applied along *c*-axis). The reference spectrum *R*_0_ is measured on the Si substrate.

We begin our investigation by confirming these basic properties of CrSBr and verifying the magneto-optical effects reported in previous studies^8,15^. An exemplary CrSBr flake used in this work, with a thickness of hundreds of nanometers, is shown in Fig. 1a. These flakes were characterized using photoluminescence (PL) and reflectance measurements performed at low temperature (5 K). The near-infrared optical spectra of each studied flake, in both PL and reflectance, exhibit several spectral features that can be identified as exciton-polaritons with energies ranging from 1.3 to 1.4 eV (see Fig. 1b), which is typical of bulk-like CrSBr flakes^16^. As demonstrated in Fig. 1c, all the observed excitonic features are linearly polarized along the *easy b*-axis of the flake, which is a consequence of the highly anisotropic crystal structure of CrSBr^8^.

By subjecting the flakes to an external magnetic field along the *c*-axis, we corroborate the strong magneto-optical effects, as presented in Fig. 1d. Upon increasing the magnetic field up to the complete interlayer FM phase transition near *B* = 2 T, the excitonic features exhibit a significant redshift of approximately 20 meV. Above the point of magnetization saturation, the field dependence becomes much weaker, unambiguously demonstrating that the observed changes originate from the interlayer antiferromagnetic ordering rather than the direct interaction of the excitons with an external magnetic field. A detailed discussion of the underlying mechanism can be found in ref. ^8^ and is also recapitulated in the following section.

### Ultra-strong magneto-electronic coupling in higher-band excitonic states

**Fig. 2:**
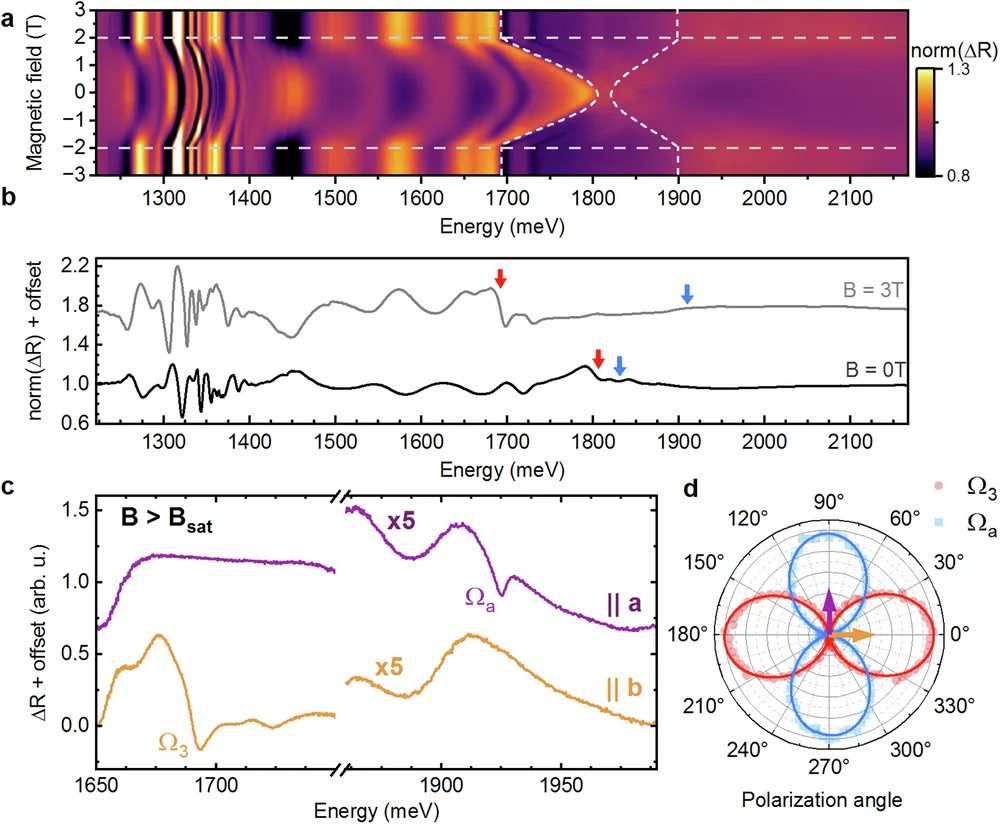
CrSBr magnetoreflectance in a wide energy range. **a**,False-color plot depicting the normalized magnetoreflectance as a function of photon energy (horizontal axis) and external magnetic field (vertical axis) applied along the c-axis of the flake, measured at the sample temperature of *T* = 5 K. The horizontal dashed lines highlight the magnetic field values at which magnetization saturates due to complete alignment of the spins with the external magnetic field. The white dashed lines serve as guides to the eye for the Ω_3_ and Ω_*a*_ excitons discussed in the text. An overlay-free version of this plot can be found in the Supplementary Material. **b** Comparison of reflectance spectra acquired in the absence of an external magnetic field and with a 3 T field applied along the *c-axis*. The arrows indicate the fitted position of Ω_3_ (red) and the approximated positions of Ω_*a*_ (blue). **c** Polarization-resolved reflectance spectra acquired with a 3 T magnetic field applied along the *c-axis* with visible features related to Ω_3_ and Ω_*a*_ transitions. **d** Anisotropy of the absorption state intensities as a function of light polarization angle relative to the *b-axis* for reported states (Ω_3_ and Ω_*a*_) marked with arrows on the spectra in panel b.

While early magneto-optical studies focused on the fundamental excitons in the range between 1.3 and 1.4 eV, numerical calculations^17,18^ predict the existence of higher-energy bands with comparable oscillator strengths. Notably, during the preparation of this paper, experimental observations of one such higher-energy band have also been reported^19–22^. To investigate such transitions, we performed a series of optical experiments over a wide energy range from 1.2 to 2.2 eV. The reflectance spectrum within this range reveals the presence of at least two additional excitonic resonances in the energy range of 1.7–1.9 eV, as can be observed in Fig. 2a and is indicated by color arrows in Fig. 2b. To unambiguously confirm the nature of these transitions, we analyzed their polarization dependence. As depicted in the polar plots in Fig. 2d, the lower of these high-energy spectral features (~1.8 eV at B = 0) exhibits the same linear polarization as the fundamental excitons, strictly aligned with the *b*-axis of the crystal, while the highest transition (~1.82 eV at B = 0) exhibits exactly perpendicular polarization.

The most striking property of these two excitonic transitions is their enormous dependence on the external magnetic field, as uncovered by our magnetoreflectance measurements (Fig. 2a). At low temperatures, the lower transition exhibits a redshift of more than 100 meV over the span of 2 T for the field along the *c*-axis. Surprisingly, upon application of a magnetic field, the higher transition is shifted towards higher energy, with the blueshift reaching Δ*E* ≈ 85 meV at *B* ≈ 2 T. To enhance the visibility of this particular transition we also prepared a sample with flakes transferred onto a CdTe-based heterostructure to provide better interference conditions. The magnetoreflectance map of that sample is included in the Supplementary Material. It is worth noting here that our analysis of the transition energies relies primarily on the energy shifts of features visible in the reflection spectra. While these shifts could in principle be affected by changes in excitonic line shapes, a more detailed examination of the reflection spectra, involving analysis of the dielectric function of the material (see Supplementary Material), reveals that the observed shifts of the reflection features are driven predominantly by excitonic energy shifts, and can thus be used—to a very good approximation—as a reliable measure of the latter. Furthermore, although cavity effects may influence the reflectance spectrum near the exciton resonances due to the high refractive index of CrSBr^15,16,19^, and in fact we do observe the characteristic additional dips in the vicinity of the first higher-energy transition that could likely be identified as distinct polaritonic branches in a thick CrSBr flake, those features shift commensurately with the main peak (similarly to the case of the rich spectrum of the fundamental excitons at 1.3–1.4 eV) and as such do not affect our analysis of the relative field-induced shifts.

The exact identity of the observed higher-energy excitons remains unknown. Theoretical studies by Qian et al.^17^ as well as by Klein et al.^18^ predict two states originating from higher bands in a similar energy range, but with no magnetic field in the simulations, the attribution of the observed peaks is only tentative. Nevertheless, in the following discussion, we will adopt the notation from ref. ^18^, denoting the fundamental excitons (in the 1.3–1.4 eV range) as Ω_1/2_, the exciton redshifted by the magnetic field from 1.8 to 1.7 eV as Ω_3_, and the exciton blueshifted by the magnetic field from 1.82 to 1.9 eV as Ω_*a*_.

**Fig. 3:**
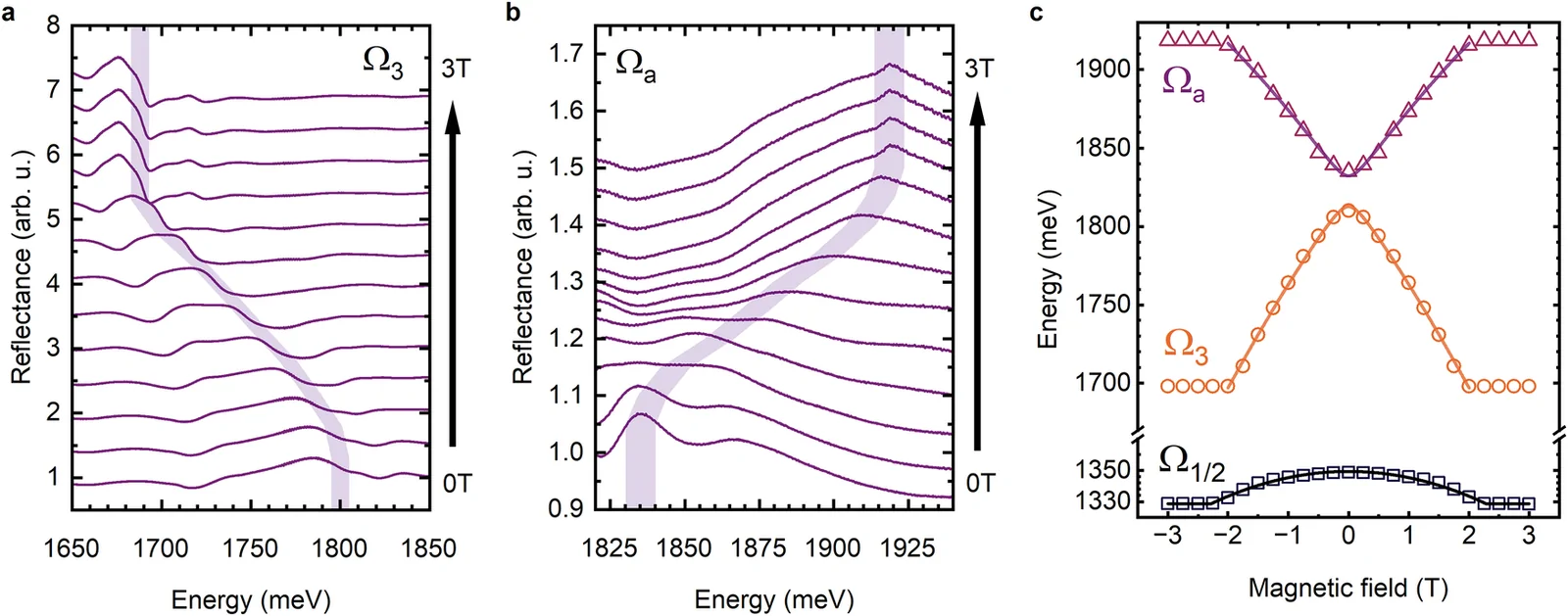
Ω_3_ and Ω_*a*_, transitions in magnetic field. **a**,**b** Magnetic field dependence of reflectance spectra in energy range corresponding to **a** Ω_3_, and **b** Ω_*a*_, transitions. Shaded areas serve as a guide to the eye indicating the position of the transition. **c** Dependence of extracted transition energy for all analyzed excitons versus magnetic field along the *c*-axis. The measurement temperature was T = 5 K. Solid lines serve as a guide to the eye.

Apart from the vastly larger energy scale and the opposite direction of the Ω_*a*_ shift, the overall shape of both shifts in the magnetic field is similar to the one observed for the fundamental excitons in 1.3–1.4 eV (see Fig. 3), thus suggesting a similar underlying mechanism. The core concept of the explanation of the Ω_1/2_ behavior is that the exciton spins are locally aligned to the magnetization of the particular monolayer^8^. At *B* = 0, the neighboring layers are oriented along the easy axis (*b*-axis), anti-parallel between the nearest-neighbor layers. Due to this anti-parallel orientation, the hybridization of the excitons between the layers does not occur. As we increase the magnetic field along the *c*-axis, the magnetization of each monolayer becomes canted toward the direction of the field. The *c*-projection of the neighboring layer magnetization follows *M* ∝ *B*_ext_, and thus the degree of interlayer hybridization (and correspondingly, lowering of the exciton energy) follows ${{\Delta }}E\propto {B}_{{{{\rm{ext}}}}}^{2}$. Around *B* = 2 T, the magnetization of each layer becomes fully oriented along the external magnetic field, and thus becomes saturated at its maximum value. Above that point, the relative magnetization between the neighboring layers does not change anymore, and the only remaining factor affecting the exciton energy is a regular excitonic Zeeman effect, much weaker than the interlayer hybridization effect described above. Such a mechanism qualitatively corresponds with the behavior illustrated in Fig. 2a, at least considering the absolute value of Ω_3_ and Ω_*a*_ energy shifts: below the transition point of 2 T the energy dependence on the magnetic field seems quadratic, and above 2 T the transition energy stays almost constant. Actually, however, the energy dependence on the magnetic field below 2 T is so strong that it slightly deviates from strictly quadratic dependence.

In order to further test the link between the layer magnetization and the behavior of Ω_3_ and Ω_*a*_ transitions, we have performed additional measurements with an external magnetic field applied along the *a*- or *b*-axis. In both cases the qualitative behavior of Ω_3_ and Ω_*a*_ was consistent with the changes reported earlier for fundamental Ω_1/2_ excitons in such field geometry^8^, with the opposite direction of the energy shift of Ω_*a*_. Applying the magnetic field along the easy axis (*b*-axis) leads to a sudden jump of the exciton energies at a field of about *B* = 0.35 T. The full magneto-reflectance false-color plots for these directions are included in the Supplementary Material.

### Effect of the temperature

**Fig. 4:**
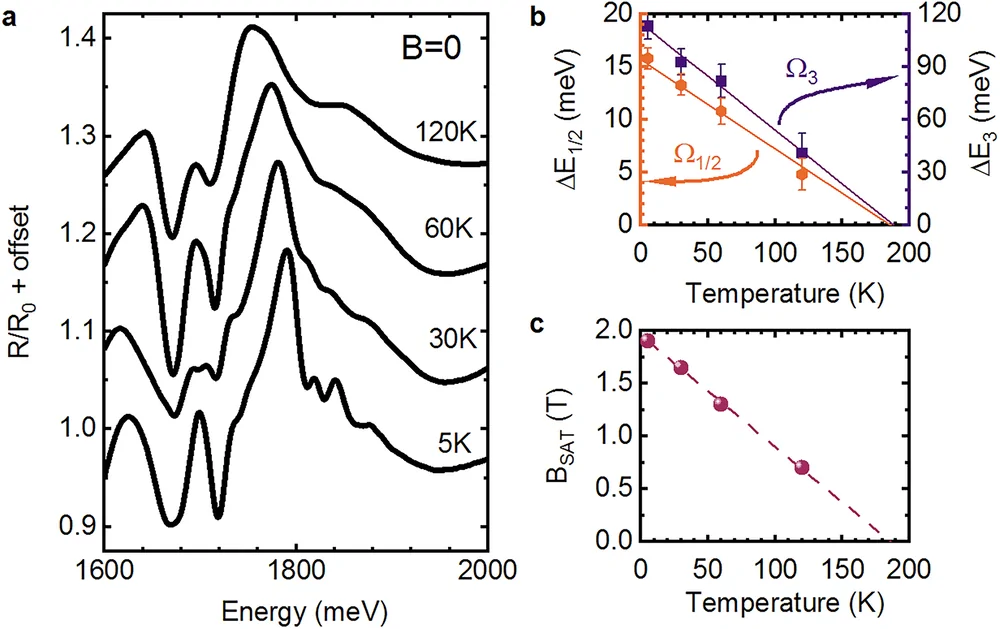
Optical transition at elevated temperatures. **a** Comparison of reflectance spectra near Ω_3_ exciton for different temperatures. **b** Total energy shifts (Δ*E*) in magnetic field along *c-axis* of Ω_1/2_ and Ω_3_ excitons versus temperature together with the linear extrapolation. **c** Magnetic field corresponding to the magnetization saturation, common for all observed excitonic states. The linear extrapolation gives critical temperature of about 185 K.

As a final experimental test of the origin of the behavior of the Ω_3_ and Ω_*a*_ resonances and their connection with the magnetic order within the crystal lattice, we harness yet another degree of freedom at our disposal: temperature. While all our results discussed so far were obtained at cryogenic conditions (*T* = 5 K), the antiferromagnetic phase of CrSBr is known to persist, albeit in a more fragile state, up to the Néel temperature of *T* = 132 K^12^. Seizing this opportunity, we perform additional magneto-reflectance measurements across an elevated temperature range. The results of this investigation are shown in Figs. 4a–c, as well as in Fig. S3 in Supplementary Material.

Upon increasing temperature, we observe gradual changes in the energy scale as well as the value of the magnetic field of the saturation point. Within experimental uncertainty, both these parameters decrease linearly with the increasing temperature, which is shown in Fig. 4b, c, respectively. It is worth noting that these changes, in particular those presented in Fig. 4b, cannot be explained by temperature-dependent excitonic energy shifts. While such shifts are common in most semiconductors, the data in Fig. 4b illustrate the *difference* between the zero-field exciton energy and the energy measured above the saturation magnetic field. The temperature-induced excitonic energy shift, primarily driven by changes in the energy band gap, should affect both these values similarly in the first approximation. Therefore, changes of the energy scale shown in Fig. 4b must be primarily driven by the magnetic properties of the material.

The linear change of both the energy scale and the value of the saturation point magnetic field stands in marked contrast to, e.g., the change of the strength of the second-harmonic generation signal in ref. ^11^, which was shown to follow ${(1-T/{T}_{c})}^{\beta }$ with critical exponent *β* = 0.36. In fact, an approximately linear temperature dependence of the energy shift has been observed for fundamental optical transitions in ref. ^15^. The authors of that work identify such temperature dependence as a magneto-optical response of the exciton energy to the population of incoherent magnons. On top of that, the critical temperature obtained by extrapolating the data in Fig. 4b, c is about 185 K, which is significantly above the Néel temperature (132 K in bulk). The temperature of 185 K extrapolated from our experiments is very close to the third critical temperature found in refs. ^15,23^, which corresponds to the intermediate ferromagnetic phase, i.e., when the interlayer antiferromagnetic coupling is already overcome by the temperature, but the intralayer ferromagnetic order is present separately in each layer. This observation is reinforced by an argument that the magnetization canting behavior is determined by the interplay between magnetocrystalline anisotropy and the external magnetic field, both of which are independent of interlayer ordering, which in turn is pertinent for A-type antiferromagnetism. The disappearance of the interlayer ordering upon approaching the Néel temperature is, however, a factor contributing to the gradual disappearance of the excitonic lines at elevated temperatures. As shown in Fig. 4a, the excitonic features in the optical spectra become significantly broader at temperatures above 100 K, which is to be expected due to increasing randomness of relative interlayer orientations. Nevertheless, the qualitatively identical behavior exhibited by both the higher-energy transition and the fundamental excitons, specifically the linear decrease in energy shift and the shared critical temperature, points once more to a common underlying origin: the magnetic ordering of the Cr atoms, rather than direct interaction with an external magnetic field.

### Theoretical perspective on magnetic-field-induced higher-band exciton shifts

As shown in Fig. 2d, the Ω_3_ and Ω_*a*_ resonances exhibit mutually orthogonal linear polarizations, aligned along the crystallographic *b*- (y) and *a*- (x) axes, respectively. This polarization selectivity is robust against an applied magnetic field. To explain this behavior and to shed some light on the possible origin of the Ω_3_ and Ω_*a*_ transitions, we carried out excitonic calculations within the TDDFT+mBSE framework (see computational details in SM), using the experimental lattice parameters of bulk CrSBr and considering two magnetic configurations: the A-type antiferromagnetic (A-AFM, *B* = 0 T) and the ferromagnetic (FM, *B* ≈ 2 T) phases. The calculated imaginary part of the dielectric function exhibits low-energy absorption peaks for *y*-polarized light, which we assign to the strongly bound fundamental excitons Ω_1/2_ with binding energy *E*_*b*_ ≈ 260 meV. At higher excitation energies, Ω_3_ emerges as the next bright *y*-polarized state with finite oscillator strength, consistent with the symmetry and energy scale of the excitonic manifold observed in magneto-optical experiments (Fig. S8 in Supplementary Material). In contrast, for *x*-polarized light the first optically allowed transition corresponds to Ω_*a*_, which lies ~20 meV above Ω_3_ and carries substantially weaker oscillator strength. Comparison of the A-AFM and FM phases confirms this identification, as we obtain qualitative agreement with the experimental trends. These results are also consistent with previous theoretical findings^17,18^. Importantly, however, all of the considered excitons are redshifted when going from the A-AFM to the FM phase, with shifts more than twice smaller than those observed experimentally. This is likely due to the shortcomings of the TDDFT+mBSE framework, which, in our case, renders it prohibitively expensive to account for spin-orbit coupling. As we show below, a single-particle approach that takes spin-orbit coupling into account can qualitatively explain both the direction and the magnitude of Ω_3_ and Ω_*a*_ shifts.

**Fig. 5:**
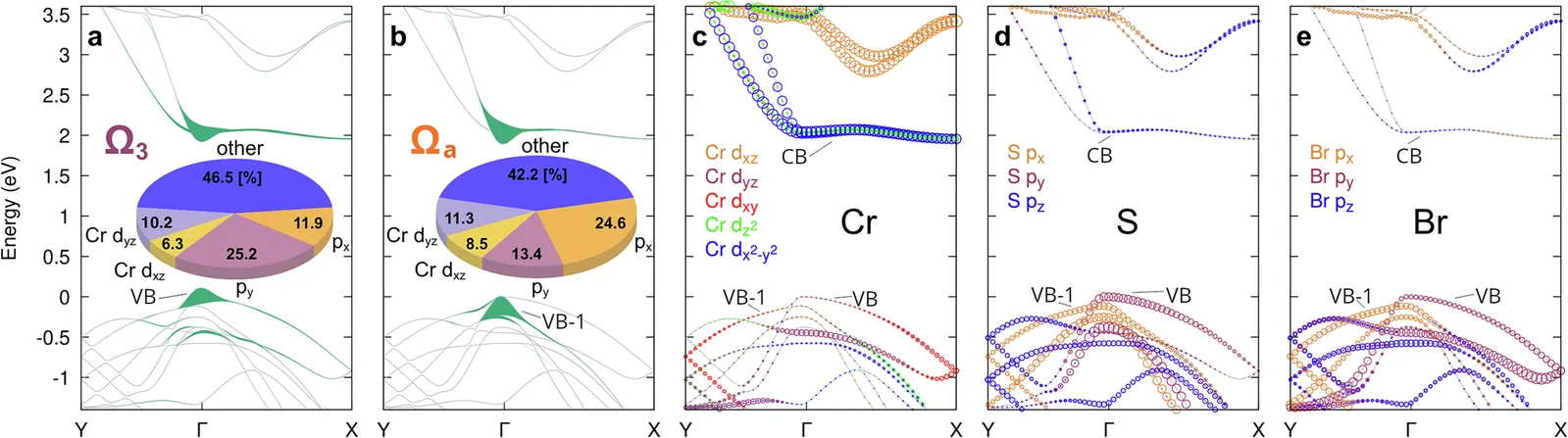
CrSBr band structure. **a**,**b** Band structure of bulk CrSBr in the A-type antiferromagnetic (A-AFM) phase, obtained using the double-screened hybrid (DSH) functional, together with the decomposition of the Ω_3_ and Ω_*a*_ excitonic wavefunctions computed within the TDDFT+mBSE framework. The corresponding insets illustrate the orbital composition of these excitonic states, while (**c–e**) present the band projections onto the Cr, S, and Br orbitals, respectively.

In order to understand the different polarization of light that is coupled to Ω_3_ and Ω_*a*_, we examine the excitonic wavefunctions and the orbital character of the bands that compose them (see Fig. 5). While the same conduction-band states have the same contributions in both excitons, the valence-band contributions are completely different (See Fig. 5). In other words, although both Ω_3_ and Ω_*a*_ share the same conduction-band character, dominated by ${d}_{{x}^{2}-{y}^{2}}$, their polarization and relative intensities are fixed by the symmetry and orientation of the valence orbitals. In orthorhombic CrSBr, the in-plane mirror operations constrain the dipole selection rules: light polarized along *y* couples to states that are odd with respect to *y*, whereas light polarized along *x* couples to states that are odd with respect to *x*. Around the relevant k-points, the conduction-band states are approximately axial in symmetry, derived mainly from ${d}_{{x}^{2}-{y}^{2}}$ and ${d}_{{z}^{2}}$, which allows them to couple to either the *p*_*y*_, *d*_*yz*_ or the *p*_*x*_, *d*_*xz*_ channels. As a result, Ω_3_ (VB,  ≈ *p*_*y*_ + *d*_*yz*_) is bright under *y*-polarization, while Ω_*a*_ (VB-1,  ≈ *p*_*x*_ + *d*_*xz*_) is bright under *x*. The markedly lower intensity of Ω_*a*_ stems from the poorer spatial overlap of *p*_*x*_, *d*_*xz*_ with the nearly axial ${d}_{{x}^{2}-{y}^{2}}$, ${d}_{{z}^{2}}$ conduction states along the *a* (*x*) direction, while the quasi-1D bonding along *b* (*y*) enhances the *p*_*y*_-to-CB overlap and velocity matrix elements for *y* polarization, consistent with the strong in-plane linear dichroism.

**Fig. 6:**
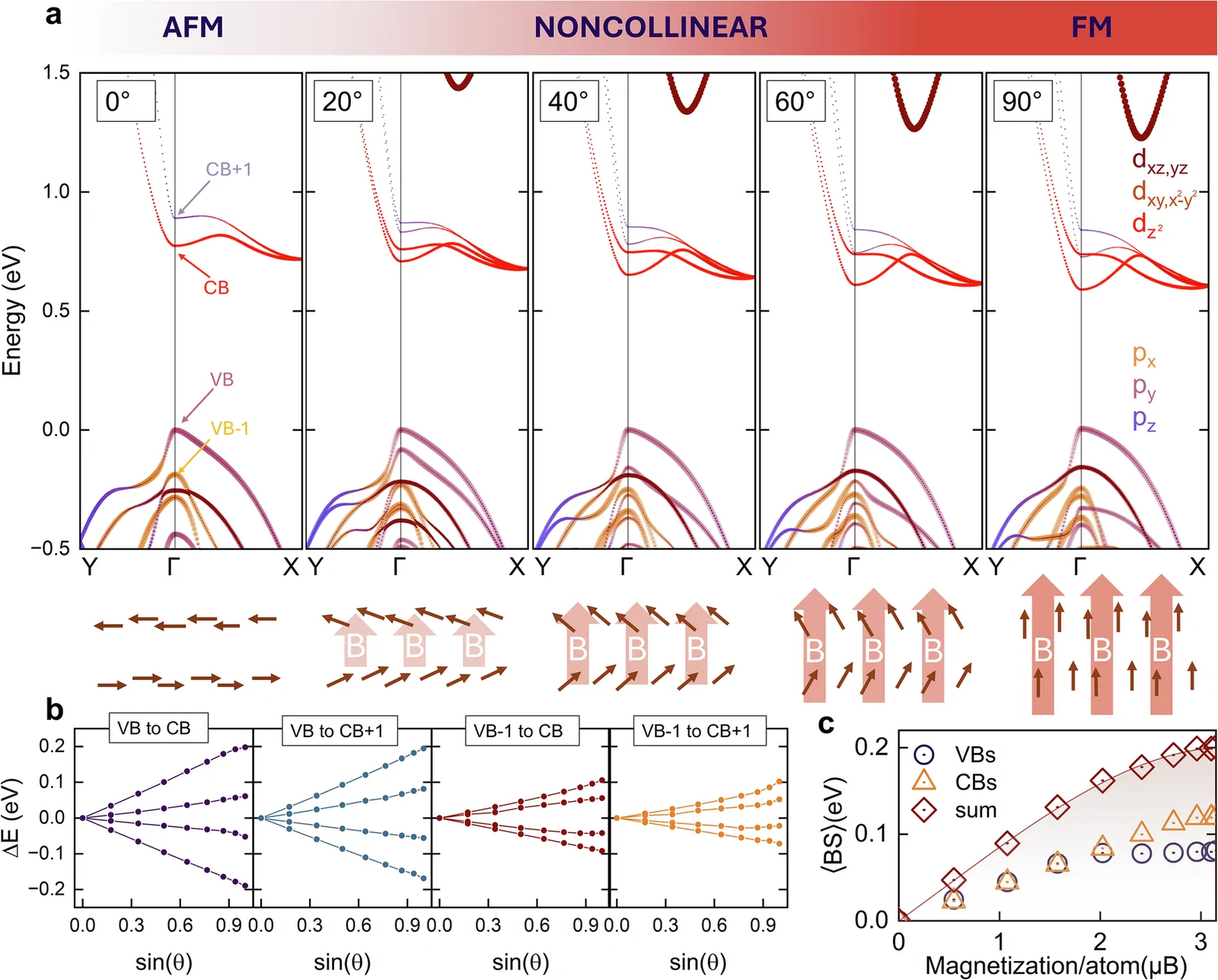
Influence of magnetic field on CrSBr band structure. **a** Evolution of the electronic band structure of bulk CrSBr under spin canting, showing orbital-resolved contributions obtained within the DFT+U+SOC framework for selected canting angles *θ*. Schematic spin configurations of adjacent layers are illustrated by brown arrows at the bottom of the panels. **b** Energy shifts Δ*E* of representative interband transitions (VB → CB, VB → CB+1, VB-1 → CB, VB-1 → CB+1) as a function of *sinθ*, showing the splitting into four branches upon canting. The Ω_3_ excitonic wavefunction is primarily composed of transitions from the valence band (VB) to the conduction band (CB) and from VB to CB+1, while Ω_*a*_ mainly arises from transitions between VB-1 and CB as well as VB-1 and CB+1. **c** Average band splittings $\left\langle E\right\rangle$ for valence bands (VBs), conduction bands (CBs), and the total sum, plotted as a function of magnetization per atom at the Γ point, calculated without including spin degeneracy for the A-AFM configuration (*θ* = 0 magnetization).

To elucidate the origin of the large magnetic field-induced shifts of Ω_3_ and Ω_*a*_, we performed non-collinear DFT+U calculations of the field-dependent band structure (Fig. 6). Rotating the Cr spin moments by an angle *θ* reflects the action of an external magnetic field and lifts the spin degeneracy of both valence and conduction bands. As shown in Fig. 6a, successive non-collinear spin configurations allow us to track the evolution of individual band energies with increasing effective field along the *z* axis. In Fig. 6b, the doubly degenerate VB/VB-1 to CB/CB+1 transitions, which host the Ω_3_ and Ω_*a*_ excitons, split into four distinct branches. Two shift upwards and two downwards, with amplitudes scaling approximately linearly with *sinθ*. The observed dependence can be understood intuitively: in the perfectly collinear AFM state (*θ* = 0) the spins are antiparallel and no transverse component exists, so no splitting occurs. As the spins begin to cant, a transverse spin component develops that is proportional to *sinθ*. This transverse part mixes states of opposite spin and drives the level repulsion responsible for the band shifts. Because the strength of this mixing grows linearly with the transverse component, the energy shifts of the bands—and hence of the excitons—increase nearly linearly with *sinθ*. At small angles the effect is weak, while at *θ* = 90 (FM limit) the transverse component is maximal and the splitting reaches its largest value. Deviations from perfect linearity originate from the multiband nature of the system and spin-orbit coupling, but the dominant trend is simply geometric: the more the spins tilt away from the *b* (*y*) axis, the larger the transverse component and the stronger the band-edge repulsion. It is important to note that Ω_3_ and Ω_*a*_ are not tied to a single interband transition or a single branch in Fig. 6a, but are built from a superposition of states across different bands and *k*-points.

The opposite net trends of Ω_3_ and Ω_*a*_ do not originate from averaging over mixed contributions, but are determined by the symmetry of the orbital channels and their polarization-dependent dipole couplings. In the VB channel (dominated by *p*_*y*_ hybridized with *d*_*yz*_), *y*-polarized light couples most strongly to the downward-moving branches. The corresponding upward partners are weak or optically dark, as their dipole matrix elements are suppressed by the phase and parity of the *p*_*y*_/*d*_*yz*_ overlap along the *b* direction. As a result, Ω_3_ predominantly follows the downward branch and exhibits a robust redshift. In contrast, the VB-1 channel (dominated by *p*_*x*_ hybridized with *d*_*xz*_) couples preferentially to *x*-polarized light, which addresses the upward-moving branches. The downward companions in this case carry little oscillator strength, so Ω_*a*_ tracks the upward branch and manifests as a blueshift. In other words, the sign of the exciton shift is dictated by orbital geometry and polarization-specific selection rules: the canting-induced transverse exchange projects differently onto the *p*_*y*_/*d*_*yz*_ and *p*_*x*_/*d*_*xz*_ manifolds in the quasi-1D crystal environment, rendering one set of branches optically dominant in Ω_3_ and the opposite set dominant in Ω_*a*_.

While these findings do not fully capture the many-body nature of Ω_3_ and Ω_*a*_, they demonstrate that the observed colossal shifts are best understood as a direct consequence of the field-driven modification of the underlying single-particle bands. Within the single-particle picture, we indeed observe pronounced canting-induced changes in both valence and conduction states that quantitatively account for the magnitude and opposite signs of Ω_3_ and Ω_*a*_. While modifications to exciton binding energies or many-body electron-hole interactions may contribute, the dominant mechanism is the exchange-driven reconstruction of the band structure itself, which evolves most strongly under spin canting.

### Summary

In summary, we have observed unprecedentedly strong magneto-optical effects in CrSBr that surpass the response of any previously studied 2D semiconductor system. By investigating the unexplored spectral range above the energy of fundamental excitonic transitions, we have demonstrated that transitions in this higher energy range exhibit a significantly enhanced sensitivity to the magnetic order. These higher energy transitions show anisotropy and gradual field dependence related to those of the fundamental excitonic transitions, but offer order-of-magnitude larger field-induced spectral shifts, approaching 100 meV. Notably, we identify two excitonic transitions, Ω_3_ and Ω_*a*_, that surprisingly exhibit field-induced shifts of opposite sign.

These experimental findings are corroborated by state-of-the-art DFT and TDDFT+mBSE calculations. Our single-particle approach (DFT+U+SOC) analysis identifies spin canting as the primary origin of the observed colossal exciton shifts. Tilting the Cr spins under an external magnetic field lifts the spin degeneracy of the valence and conduction states and reconstructs the band edges nearly linearly with $\sin \theta$, the transverse spin component. This reconstruction produces large and opposite shifts of the Ω_3_ and Ω_*a*_ excitons: Ω_3_ follows the downward-moving *p*_*y*_/*d*_*yz*_ branches addressed by y-polarized light and redshifts, while Ω_*a*_ follows the upward-moving *p*_*x*_/*d*_*xz*_ branches addressed by x-polarized light and blueshifts. The sign and magnitude of the shifts are therefore fixed by orbital geometry and polarization-dependent dipole selection rules. Although many-body effects not captured by our theoretical model—such as changes in exciton binding energy—may play a role, the dominant mechanism is the exchange-driven reconstruction of the single-particle band structure under spin canting, which naturally accounts for both the magnitude and the contrasting trends of Ω_3_ and Ω_*a*_. We hope that our theoretical findings will stimulate further discussion on the unusual behavior of those higher-energy transitions, and fuel future, more refined theoretical work that, for example, fully accounts for spin-orbit coupling beyond the independent-particle approximation.

The discovery of such colossal magneto-optical effects in CrSBr represents a significant advancement in the understanding of magneto-optical coupling in 2D magnetic semiconductors and its exploration with unprecedented sensitivity levels, overcoming limitations imposed by the relatively weak magnetic responses in conventional systems.

## Methods

### Sample preparation

The sample used in this work was prepared through mechanical exfoliation of bulk CrSBr material onto a Si (111) substrate. The sample contains a large number of rectangular flakes of various thicknesses, in the range of hundreds of nanometers. Several of those flakes were randomly selected for magneto-optical measurements.

### Measurement technique

To characterize the CrSBr flakes using PL and reflectance measurements at cryogenic temperatures, the sample was placed inside a liquid-He bath cryostat equipped with Variable Temperature Insert (VTI), a vector-rotate magnet (i.e., two sets of superconducting magnet coils in a perpendicular configuration), and four optical windows. This setup allowed for the application of a magnetic field of up to 3 T in any direction with respect to the sample’s axes, as well as control of the sample’s temperature in the range from 4 K to room temperature. The optical signal from the sample was recorded with a Czerny-Turner spectrometer equipped with a CCD camera.

### DFT calculations

To reproduce the optical spectra for the A-AFM (*B* = 0 T) and FM (*B* ≈ 2 T) configurations and to capture excitonic effects arising from electron–hole interactions, we employed a two-step computational strategy combining ground-state hybrid-functional calculations with excited-state TDDFT+mBSE analysis. A fully rigorous treatment of excitonic behavior in the presence of a moderate external field would require solving the BSE with explicit inclusion of non-collinear magnetization and spin-orbit coupling (SOC). Such calculations are computationally demanding for CrSBr, owing to its large unit cell and the necessity of dense Brillouin-zone sampling. Therefore, to elucidate the origin of the large magnetic-field-induced shifts of higher-energy excitonic transitions, we instead adopted a simplified single-particle approach: non-collinear DFT+U+SOC calculations of the field-dependent band structure. Further computational details are provided in the Supplementary Material.

## Supplementary information

Supplementary Information

Transparent Peer Review file

## Data Availability

All data generated in this study are provided within the paper, its Supplementary Material and in the Figshare database (https://doi.org/10.6084/m9.figshare.30649298).
