# Supplementary material for: Colossal magnetic tunability of excitons in 2D van der Waals magnetic semiconductor CrSBr
Source: Nat Commun. 2026 Sep 24;17:11096. doi: 10.1038/s41467-026-77986-z (PMC13696676; doi:10.1038/s41467-026-77986-z)
Supplement: Supplementary file 1 [file 41467_2026_77986_MOESM1_ESM.pdf]

Colossal magnetic tunability of excitons in 2D van der Waals magnetic semiconductor CrSBr – Supplementary Material

R. Komar[1], M. Goryca[1,*], A. Łopion[1,2], M. Rybak[3], T. Woźniak[3,4], M. Raczyński[1], K. M. Gałazka[1], K. Mosina[4], A. Söll[4], Z. Sofer[4], W. Pacuski[1], C. Faugeras[5], M. Birowska[1], P. Kossacki[1,], and T. Kazimierczuk[1]**

[1]Institute of Experimental Physics, Faculty of Physics, University of Warsaw, Warsaw, 02-093 Poland
[2]Institute of Physics, University of Muenster, Muenster, 48149, Germany
[3]Faculty of Fundamental Problems of Technology, Wrocław University of Science and Technology, Wrocław, 50-370 Poland
[4]Institute of Theoretical Physics, Faculty of Physics, University of Warsaw, Warsaw, 02-093 Poland
[5]Department of Inorganic Chemistry, University of Chemistry and Technology Prague, Technicka 5, 166 28 Prague 6, Czech Republic
[6]LNCMI, UPR 3228, CNRS, EMFL, Université Grenoble Alpes, 38000 Grenoble, France
[*]mgoryca@fuw.edu.pl
[**]Piotr.Kossacki@fuw.edu.pl

ABSTRACT

This document contains supplementary materials for the article "Colossal magneto-excitonic effects in 2D van der Waals magnetic semiconductor CrSBr"

1 Analysis of CrSBr dielectric function

Our analysis of the energy shifts of observed transitions is based mainly on the analysis of the energy shifts of the features visible in the reflection spectra. However, those shifts might also be affected by the changes in the excitonic line shapes. In order to validate the analysis based on reflection spectra, we examine those spectra in more detail, which involves analysis of the dielectric function of the material. Fig. S1a shows the dielectric function resulting from fitting the experimental zero-field reflection spectrum with a calculated one, using the Ω_1 and Ω_3 exciton oscillator strength, exciton energy, and damping as fitting parameters (Fig. S1b). Figs. S1c-d show the the same for the $B > B_{sat}$ case. Obtained energy shifts of the fitted transitions closely correspond (within 10% error bar) with the experimental shifts from the reflection spectra. Therefore, the shifts of the reflection features observed in the experiment are primarily driven by the shifts of the exciton energies and as such can be used with very good approximation as a measure of the latter.

2 Magnetoreflectance on a different substrate

The magnetoreflectance measurements were repeated on another set of CrSBr flakes that were exfoliated on a sample containing a (Cd,Mn)Te quantum well in (Cd,Mg)Te barriers. Such substrate provided different conditions of optical interference. Obtained results in the spectral range corresponding to Ω_3 and Ω_a excitons are shown in Fig. S2.

3 Magnetoreflectance at elevated temperature

The magnetoreflectance measurements of CrSBr flakes on Si substrate were also performed at elevated temperature. An example result obtained at $T = 60$K is shown in Fig. S3.

4 Plot of magnetoreflectance without overlay

For the sake of readability, the false-color plot depicting the magnetoreflectance shown in Figure 2 in the main article is presented with a dashed-line overlay, serving as guide for an eye. The same plot but without the overlay is shown in Figure S4.

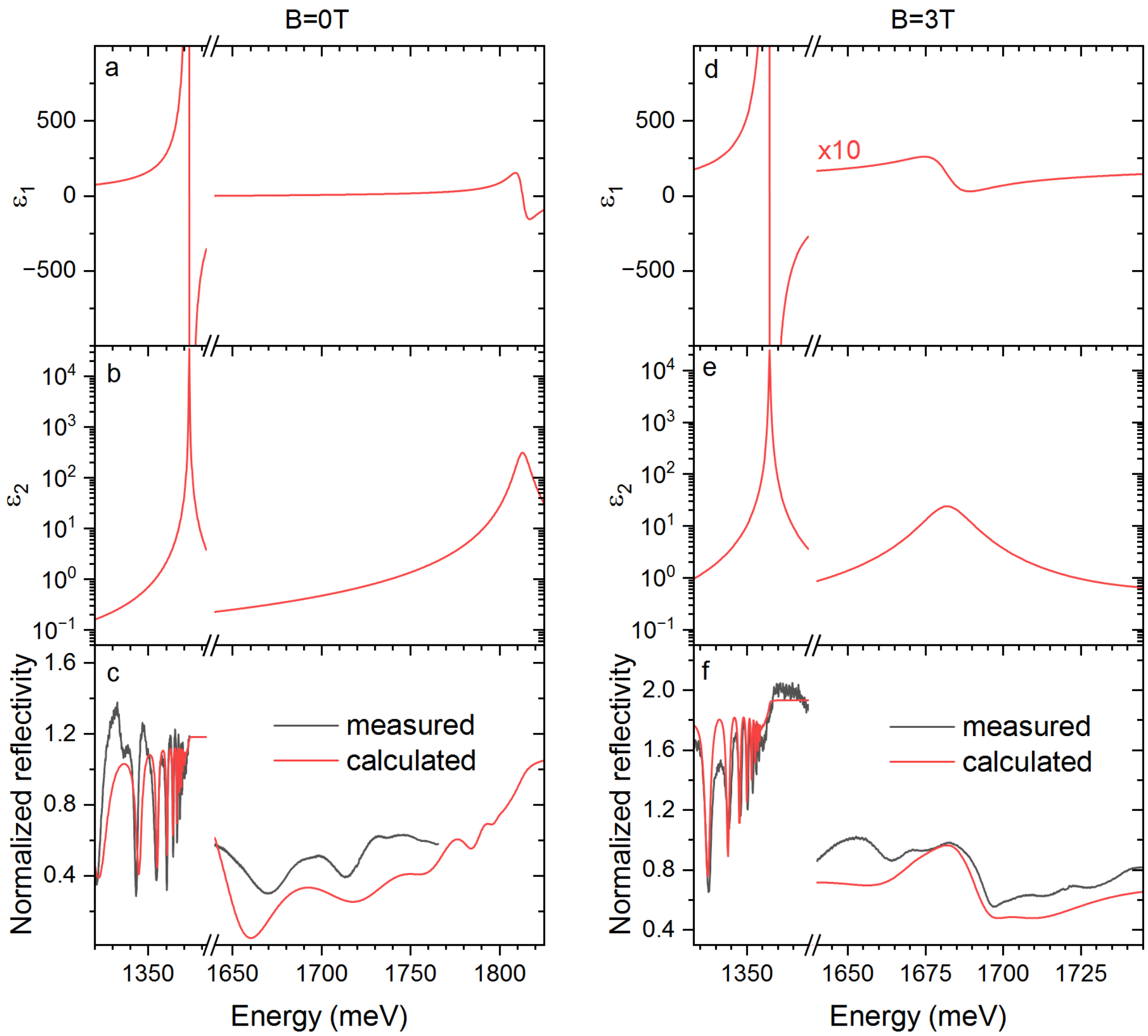

Figure S1. Real (**a**, **d**) and imaginary (**b**, **e**) part of CrSBr dielectric function at $B = 0\,\mathrm{T}$ and $B = 3\,\mathrm{T}$, respectively, resulting from fitting the experimental reflection spectra with calculated ones (**c**, **f**). The fitted values of the transition energies are: 1373.0 eV and 1813.0 eV at $B = 0$; 1357.4 eV and 1682.0 eV at $B = 3\,\mathrm{T}$.

5 Anisotropy of magnetic properties

CrSBr crystal has an orthorhombic symmetry[1] with three non-equivalent axes. The core findings of our work are related to experimental configuration with external magnetic field oriented along *c-axis*, i.e., perpendicularly to the exfoliated layers. With regard to magnetic properties, this is the hard-axis direction. Figures S5 and S6 present the analogous data measured for magnetic field oriented along the (intermediate) *a-axis* (easy) *b-axis*, respectively.

6 Computational Details

6.1 Excitonic calculations (TDDFT+mBSE)

To accurately reproduce the optical spectra and capture excitonic effects arising from electron–hole interactions, we employed a two-step computational strategy combining ground-state hybrid-functional calculations with excited-state TDDFT+mBSE analysis, as implemented in VASP (version 6.4.3)[2].

Ground-State Calculations. The ground-state electronic structure was obtained within the projector augmented-wave (PAW) formalism[?] using the Perdew–Burke–Ernzerhof (PBE) exchange–correlation functional[?,2]. Experimental lattice parameters were adopted ($a = 3.5761$ Å, $b = 4.7859$ Å, $c = 7.9838$ Å)[?]. To describe the A-type antiferromagnetic (A-AFM) stacking of layers, the out-of-plane lattice vector was doubled, ensuring alternating spin alignment between adjacent layers. Atomic coordinates were relaxed until residual Hellmann–Feynman forces were below 10^{-3} eV Å^{-1}, while keeping the lattice constants fixed. The plane-wave kinetic energy cutoff was set to 450 eV, and the Brillouin zone was sampled with a

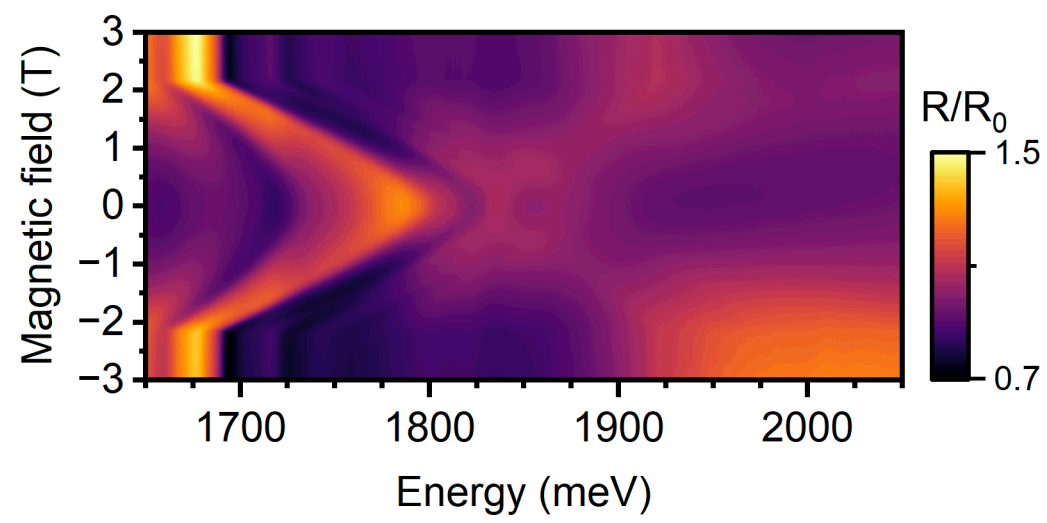

Figure S2. False-color plot depicting the magnetoreflectance as a function of photon energy (horizontal axis) and external magnetic field (vertical axis) applied along the*c-axis* of a CrSBr flake on a (Cd,Mg)Te/(Cd,Mn)Te/(Cd,Mg)Te substrate.

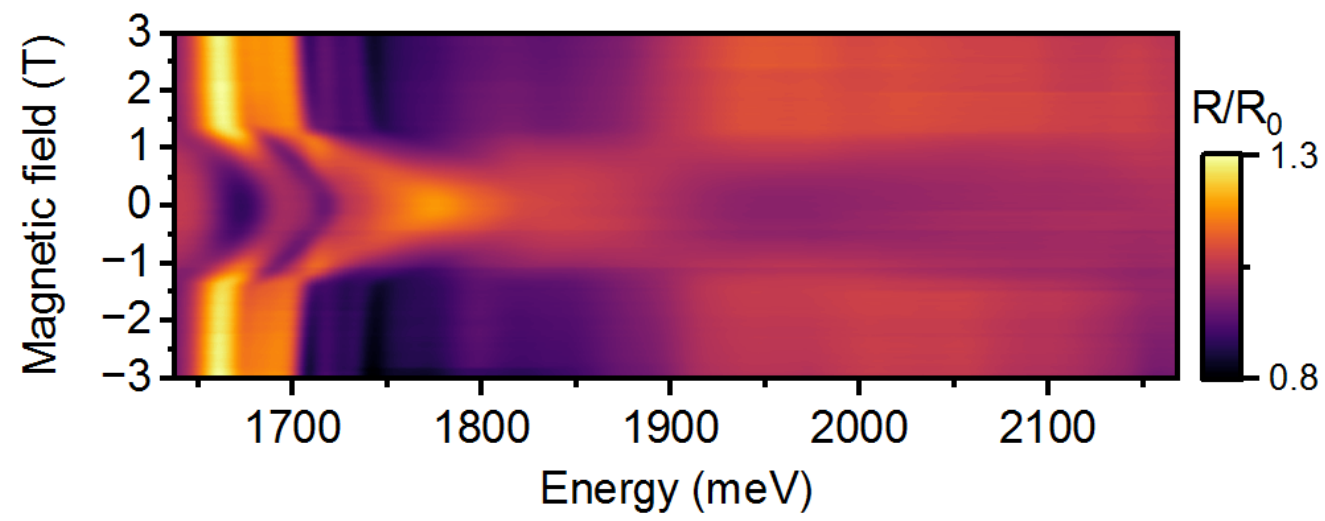

Figure S3. False-color plot depicting the magnetoreflectance as a function of photon energy (horizontal axis) and external magnetic field (vertical axis) applied along the*c-axis* of a CrSBr flake on Si substrate obtaned at $T = 60$K.

$16 \times 12 \times 4$ Monkhorst–Pack grid. Electronic occupations were treated with a Gaussian smearing of 0.05 eV, and the total energy convergence threshold was 10^{-7} eV. These parameters ensured fully converged total energies and forces, and provided sufficiently accurate Kohn–Sham wavefunctions for subsequent evaluation of optical transition matrix elements.

Double-Screened Hybrid (DSH) Functional. To obtain an improved description of the electronic band structure and to reproduce quasiparticle-like band gaps, the double-screened hybrid (DSH) functional[?] was employed. The DSH approach extends conventional hybrid functionals by incorporating a physically motivated, dielectric-dependent screening of the Fock exchange interaction. In this framework, the Coulomb potential is separated into short- and long-range components, where the range-separation parameter μ defines the spatial extent of the screened exchange. Each part of the exchange interaction is then weighted by an effective screening determined from the material's electronic dielectric response. The short-range and long-range contributions are screened differently, reflecting local and macroscopic polarization effects, respectively. Two parameters govern the DSH functional[?]: (i) the fraction of long-range exact exchange, β, and (ii) the range-separation parameter, μ. In the self-consistent DSH procedure implemented in VASP, these parameters are derived from the system's own electronic screening without any empirical fitting. Specifically, the macroscopic dielectric constant ε_∞ is first evaluated within a model random-phase approximation, and the long-range exchange fraction is set to $\beta = 1/\varepsilon_\infty$. The parameter μ is subsequently obtained by fitting a model dielectric function, which describes the momentum-dependent screening of the Coulomb interaction. This procedure yields a fully self-consistent dielectric-dependent hybrid functional, where the amount and range of exact exchange are directly linked to the material's intrinsic screening properties. In practice, the self-consistent determination of β and μ was performed separately for the antiferromagnetic (AFM) and ferromagnetic (FM) configurations, resulting in $\beta_{\mathrm{AFM/FM}} = 0.1545/0.1561$ and $\mu_{\mathrm{AFM/FM}} = 2.8040/2.7446$ Å^{-1}. These values correspond to physically reasonable dielectric constants consistent with moderately screened exchange. To ensure quantitative agreement with experiment, the final DSH parameters were slightly adjusted so that the resulting DSH band gaps reproduced the experimental quasiparticle band gaps extracted from angle-resolved photoemission spectroscopy (ARPES)[?]. The resulting DSH-corrected eigenvalues and wavefunctions were subsequently used as input for the optical and excitonic calculations described below.

Excitonic and Optical Calculations The optical response was computed within time-dependent density functional theory (TDDFT) using the model Bethe–Salpeter equation (mBSE) formalism in the Tamm–Dancoff approximation (TDA), as implemented in VASP. This approach includes both the screened electron–hole attraction and the exchange-type local-field effects, thus accounting for excitonic contributions to the dielectric function. The mBSE Hamiltonian was constructed using 20 occupied and 4 unoccupied bands, while a total of 120 bands were included in the dielectric-function convergence tests. A complex shift of 0.001 eV was applied to simulate Lorentzian broadening of the spectral features in $\mathrm{Im}[\varepsilon(\omega)]$. Because full excitonic calculations for arbitrary magnetic canting angles (θ) are computationally demanding, the TDDFT+mBSE analysis

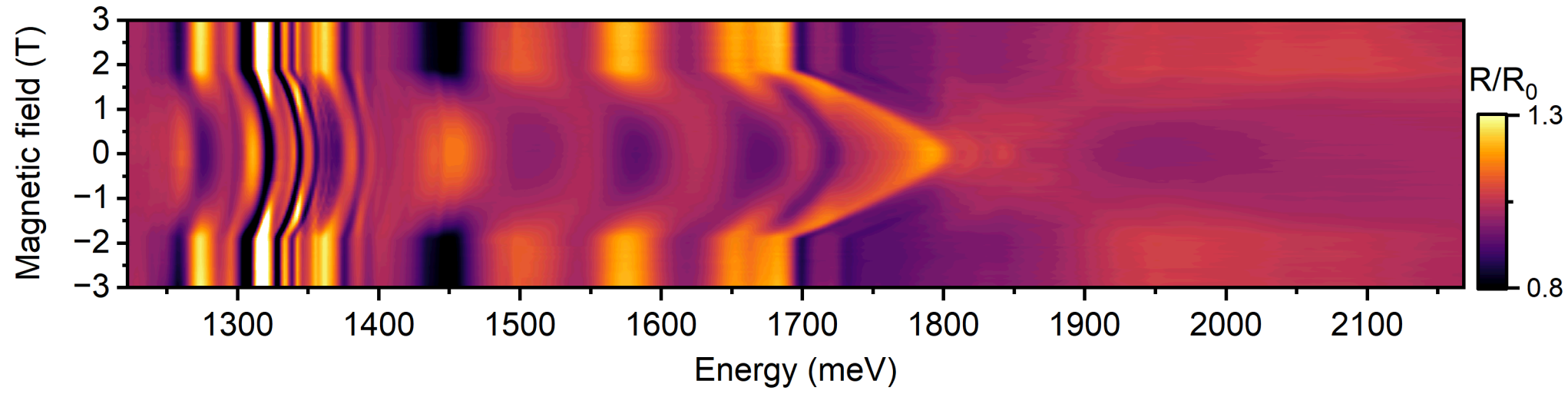

Figure S4. False-color plot depicting the magnetoreflectance as a function of photon energy (horizontal axis) and external magnetic field (vertical axis) applied along the*c-axis* of a CrSBr flake on a Si substrate.

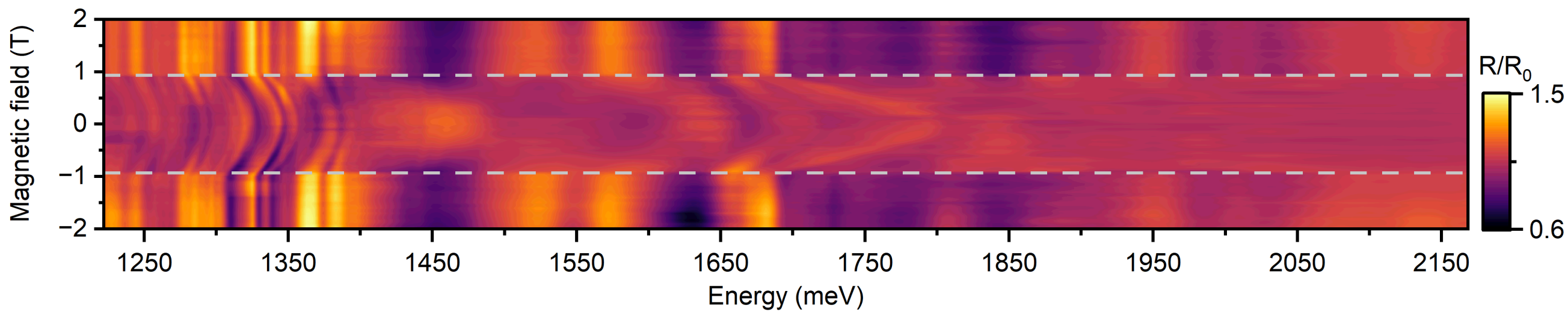

Figure S5. Magnetoreflectance map with external magnetic field along *a-axis*.

was restricted to the AFM ($B = 0$ T) and FM ($B = 2$ T) configurations, each treated in a fully spin-polarized framework to capture magnetically induced optical anisotropy.

The electronic band structure obtained using the double-screened hybrid (DSH) functional is shown in Fig.S7, together with the excitonic wavefunctions projected onto the band states to visualize the dominant valence–conduction transitions contributing to the bright ex citations: $\Omega_{1/2}$, Ω_3, and Ω_a. The optical absorption spectra, calculated within the TDDFT+mBSE framework for thw magnetic phases A-AFM and FM, are shown in Fig.S8. The absorption intensity is proportional to the imaginary part of the macroscopic dielectric function, highlighting the excitonic features and their dependence on magnetic ordering.

Importantly, all considered excitons red-shift when going from A-AFM to FM, but the calculated shifts are more than two times smaller than in experiment. Given that these shifts are only a few tens of meV, they lie within the sensitivity of our workflow to technical parameters such as the lattice constants, k-mesh density, dielectric screening (β, μ), and the size of the BSE subspace. Consequently, within our present settings, the precise sign and magnitude of the shift cannot be regarded as conclusive.

6.2 DFT+U calculations

A fully rigorous treatment of excitonic behavior in the presence of an external magnetic field would require solving the Bethe–Salpeter equation (BSE) with explicit inclusion of noncollinear magnetization and spin–orbit coupling (SOC). Such calculations are computationally demanding for CrSBr due to its large unit cell and the necessity of dense Brillouin-zone sampling. Instead, we adopt a simplified yet physically justified approach: density functional theory plus Hubbard U (DFT+U) calculations including SOC were performed for both the A-type antiferromagnetic (A-AFM, $B = 0$ T) and ferromagnetic (FM, $B \approx 2$ T) configurations. The underlying reason is that the dominant magnetic-field dependence of the excitonic spectrum arises

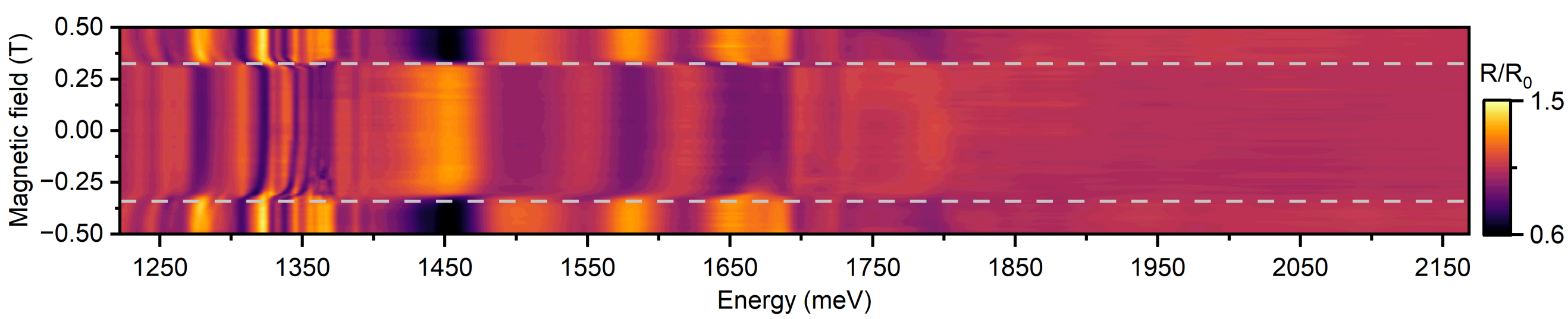

Figure S6. Magnetoreflectance map with external magnetic field along *b-axis*.

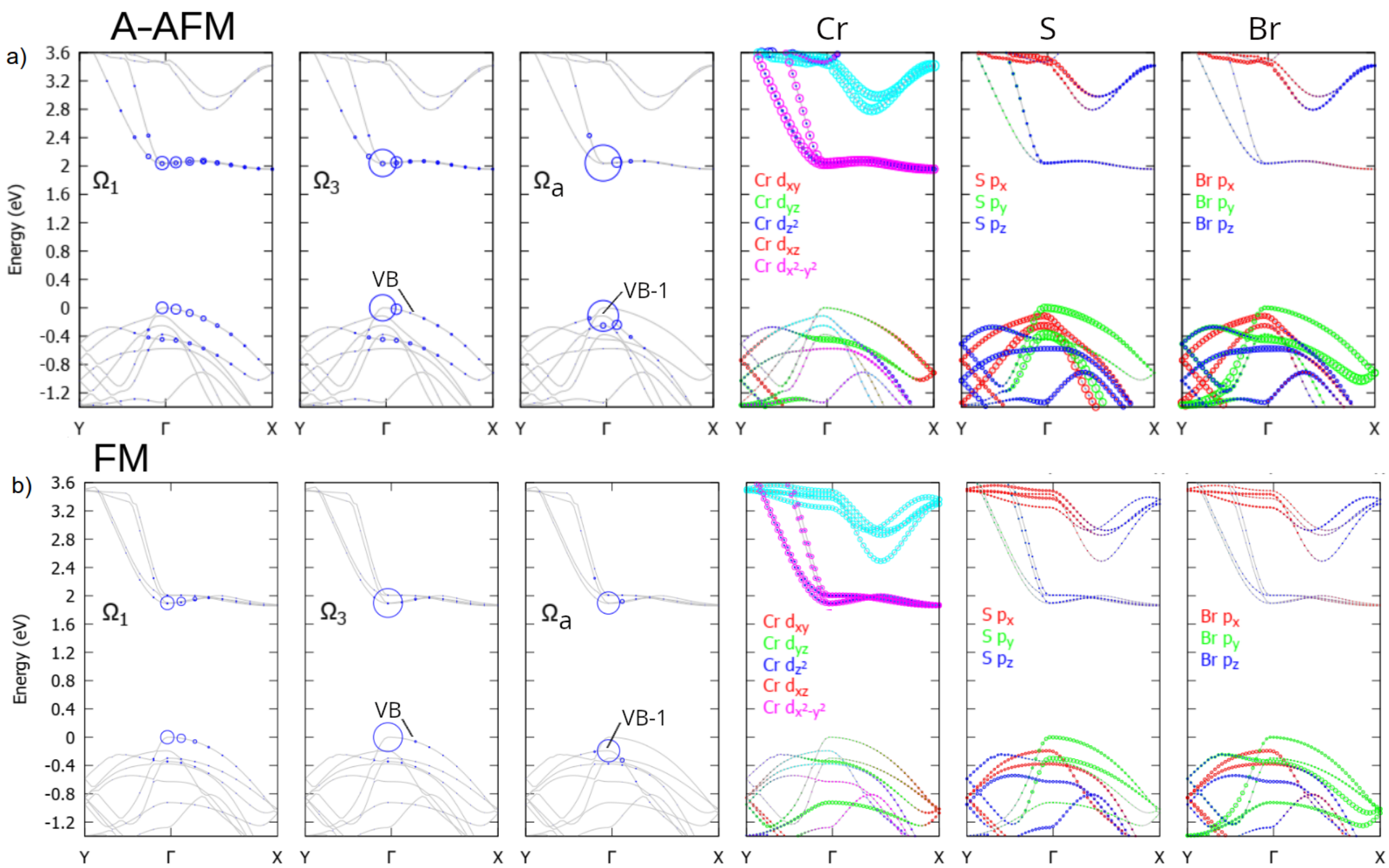

Figure S7. Band structures obtained using the double-screened hybrid (DSH) functional and excitonic properties calculated within the time-dependent density functional theory combined with the model Bethe–Salpeter equation (TDDFT+mBSE) framework. (a,b) The first three panels from the left display the excitonic wavefunctions corresponding to the first bright excitonic states (from left to right: $\Omega_{1/2}$, Ω_3, and Ω_a), projected onto the band states. The size of each circle represents the magnitude of the corresponding contribution to the excitonic wavefunction. The last three panels show the orbital-resolved band projections for Cr, S, and Br atomic states. Panels (a) and (b) correspond to the A-type antiferromagnetic (A-AFM) and ferromagnetic (FM) phases, respectively, both calculated within the spin-polarized framework.

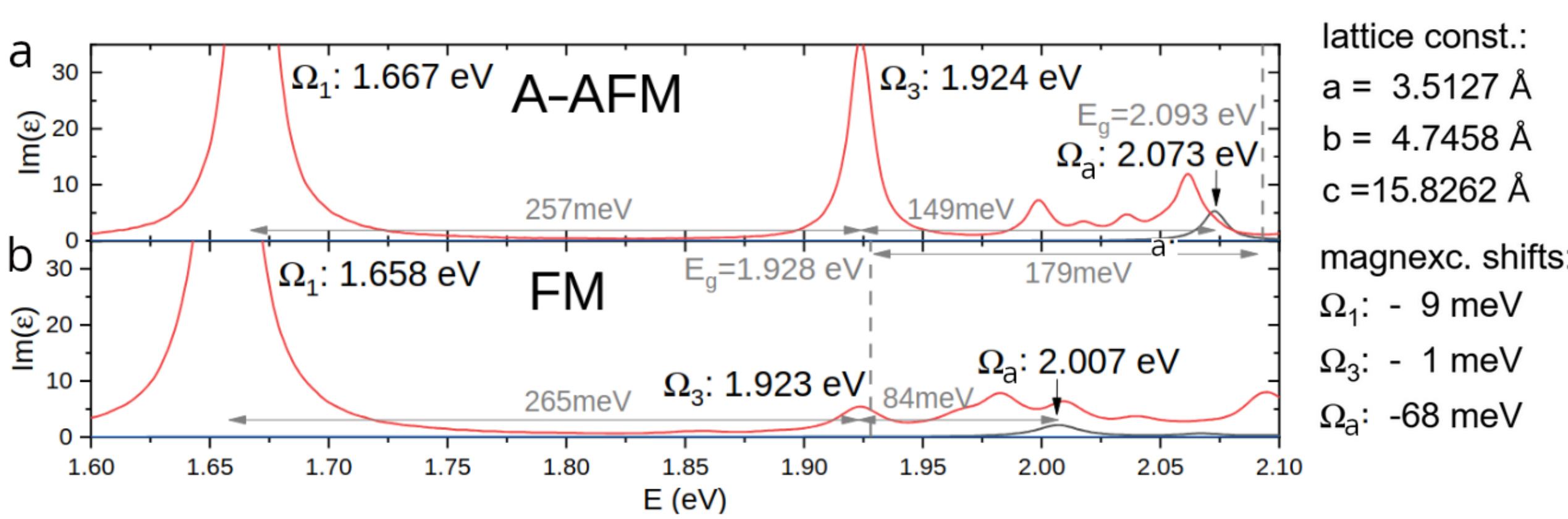

Figure S8. Imaginary part of the dielectric function for two magnetic orderings of bulk CrSBr: (a) A-type antiferromagnetic (A-AFM) and (b) ferromagnetic (FM) phases. The spectra were obtained within time-dependent density functional theory combined with the model Bethe–Salpeter equation (TDDFT+mBSE), based on the experimental lattice parameters. The right panel shows the energy shifts associated with the transition from the A-AFM to the FM phase. Red and yellow colors indicate the light polarization coupled to the *y* and *x* directions, respectively.

from modifications to the electronic band structure—specifically, shifts in band energies and corresponding changes in orbital

character. Notably, the excitonic resonances Ω_3 and Ω_a exhibit mutually orthogonal polarizations, originating from the distinct orbital compositions of the electronic bands that contribute to their respective excitonic wavefunctions. This relationship enables the identification and tracking of the electronic bands associated with specific polarization channels.

The calculations have been conducted within density functional theory (DFT) using the generalized gradient approximation as implemented in VASP software[2]. The ion–electron interactions have been described by the projector augmented wave (PAW) method[3]. Plane-wave basis cutoff and Γ centered Monkhorst-Pack[4] k-point grid have been set to 500 eV and $10 \times 6 \times 1$, respectively. A Gaussian smearing of 0.05 eV was employed for the Brillouin zone (BZ) integration. The interlayer vdW forces have been treated within Grimme scheme[5] using D3 correction[6]. The results were obtained using PBE+U method based on Dudarev's approach[7], with the effective on-site Hubbard U parameter ($U_{\mathrm{eff}} = U - J$, where J has been fixed to $J = 1$ eV) assumed for $3d$ orbitals. Note that U_{eff} is hereafter denoted as U. The impact of U parameter on electronic transitions have been previously considered in paper[8]. The SOC within the non-collinear treatment of magnetism has been taken into account on the top of the PBE+U scheme. The position of the atoms and unit cell have been fully optimized within the PBE+U approach for magnetic ground state (A-AFM). At each employed θ angle the atomic positions and the lattice parameters have been fixed to optimal position of magnetic ground state. The direct interband momentum matrix elements were computed from the wave function derivatives using Density Functional Perturbation Theory (DFPT)[9] in order to determine the transition dipole strength, as discussed in 10.

References

1. Lee, K. *et al.* Magnetic order and symmetry in the 2D semiconductor CrSBr. *Nano Lett.* **21**, 3511–3517, (2021).
2. Kresse, G. & Furthmüller, J. Efficiency of ab-initio total energy calculations for metals and semiconductors using a plane-wave basis set. *Comput. Mater. Sci.* **6**, 15–50, (1996).
3. Holzwarth, N. A. W., Tackett, A. R. & Matthews, G. E. A projector augmented wave (paw) code for electronic structure calculations, part i: atom paw for generating atom-centered functions. *Comput. Phys. Comm.* **135**, 329–347, (2001).
4. Monkhorst, H. J. & Pack, J. D. Special points for brillouin-zone integrations. *Phys. Rev. B* **13**, 5188–5192, (1976).
5. Grimme, S. Semiempirical GGA-type density functional constructed with a long-range dispersion correction. *J. Comput. Chem.* **27**, 1787 – 1799, (2006).
6. Grimme, S., Antony, J., Ehrlich, S. & Krieg, H. A consistent and accurate ab initio parametrization of density functional dispersion correction (DFT-D) for the 94 elements H-Pu. *J. Chem. Phys.* **132**, 154104, (2010).
7. Dudarev, S. L., Botton, G. A., Savrasov, S. Y., Humphreys, C. J. & Sutton, A. P. Electron-energy-loss spectra and the structural stability of nickel oxide: An lsda+u study. *Phys. Rev. B* **57**, 1505–1509, (1998).
8. Linhart, W. M. *et al.* Optical markers of magnetic phase transition in crsbr. *J. Mater. Chem. C* **11**, 8423–8430, (2023).
9. Gajdoš, M., Hummer, K., Kresse, G., Furthmüller, J. & Bechstedt, F. Linear optical properties in the projector-augmented wave methodology. *Phys. Rev. B* **73**, 045112, (2006).
10. Woźniak, T., Faria Junior, P. E., Seifert, G., Chaves, A. & Kunstmann, J. Exciton *g* factors of van der Waals heterostructures from first-principles calculations. *Phys. Rev. B* **101**, 235408, (2020).
