# Supplementary material for: Colossal magnetic tunability of excitons in 2D van der Waals magnetic semiconductor CrSBr
Source: Nat Commun. 2026 Sep 24;17:11096. doi: 10.1038/s41467-026-77986-z (PMC13696676; doi:10.1038/s41467-026-77986-z)
Supplement: Supplementary file 2 [file 41467_2026_77986_MOESM2_ESM.pdf]

nature portfolio

Peer Review File

Colossal magnetic tunability of excitons in 2D van der Waals magnetic semiconductor CrSBr

Corresponding Author: Dr Mateusz Goryca

This file contains all reviewer reports in order by version, followed by all author rebuttals in order by version.

Version 0:

Reviewer comments:

Reviewer #1

(Remarks to the Author)
This manuscript reports a large magnetic field dependent spectral shift in bulk CrSBr, which is attributed to colossal magneto-excitonic effects. The work first started with spectroscopic study of some energy states under magnetic fields. And it then tried to correlate the origins of this strong magnetic field dependence with the direct inter-band transitions based on the DFT calculations. Though the strong magnetic field dependence is very interesting, the hand-waving explanations and vague reasoning with DFT calculations made this work look premature. I cannot recommend the publication of this work at the current stage. Before further consideration, here are some comments as below.

1) The authors have performed high throughput calculations to show the strong spin-orientation dependence of various inter-band transitions (Fig. 4). Though all these transitions show strong magnetic field dependences, the vague assignments of these transitions to the contributions of the observed spectral states are confusing. The qualitative comparisons of calculated inter-band transitions with experimental observations are rough, making the validity of their calculations questionable. The hand-waving explanations with the exciton binding energies is premature. Without careful examination between experiments and calculations, the reasoning of DFT theory is not convincing.

2) The phenomenological descriptions of the magnetic field dependences in Equation (1) make the understanding of these origins difficult. Why is the field-induced coupling a valid way to describe the magnetic field dependence? Without clarifications on the physical origins, this description is just a mathematical trick.

3) How can the authors make sure that Omega_3 and Omega-4 are excitonic states with large binding energies? Based on the limited experimental data, it can be only inferred these are high-lying excited electronic states. There are no other spectral evidences (like PL, ultrafast dynamics or nonlinear optics) to support the excitonic nature of these two states. The claim of “magneto-exciton effect” is bold, and maybe misleading.

4) The anisotropic axis In Fig. 2d seems to be not along that axis of Fig. 2c. Why is there some angular difference? Does this mean these two states have different anisotropic origins?

5) Fig. 4c has very low image resolutions. The label text in these subfigures cannot be resolved.

6) In Fig. 5b, what does the linear temperature dependence of the energy shifts mean for the physical origins? The authors just showed the linear dependence without any discussion on the physical origins. For Fig. 5b and 5c, extrapolating the linear dependence is taken to infer the ferromagnetic critical temperature. This does not seem to be a valid way, as the temperature-dependent excitonic energy shifts are common and prominent for most semiconductors (even though they are not magnets).

Reviewer #2

(Remarks to the Author)
R. Komer studied the reflection spectra of CrSBr under magnetic fields. In the reflection spectra, a peak is significantly red-shifted under fields. This shift is attributed to the colossal magneto-excitonic effects. In addition, DFT simulations were performed to elaborate the experimental spectra. The results are interesting. The paper can be published if the following

issues are properly addressed.
1) The presented results are reflection contact rather than dielectric function or optical conductivity. This format could lead to misinterpretation of the exciton response. The reflection is governed by the dielectric function of CrSBr, which is determined by the exciton oscillator strength, exciton energy, and damping. All of these parameters could shift the reflection feature. However, the authors attributed the shift solely to exciton energy, without considering other potential mechanisms, such as variation of oscillator strength and change of the exciton line shape. In order to address this concern, which is essential to the key conclusion of this work, the author should extract the optical conductivity or dielectric function. Then, the extracted exciton energies as a function of the magnetic field should be used to illustrate the exciton shift.

2) There are multiple peaks in the PL spectra whose origin and correspondence to the reflection spectra are not discussed. They could arise from the Fabry-Perot cavity formed by crystal flakes.
The cavity geometry also complicates the reflection spectra, indicating the need to analyze the dielectric function or optical conductivity.

3) In fig. 2a, the reflection is enhanced by magnetic fields in the range of 1.9 eV~2 eV. What is the origin of this enhancement? In addition, it seems that the data is overly smoothed., how many spectra are plotted in fig.2a? Raw data may be added to the supplementary materials without any process.

4) The authors investigated the temperature dependence of spectra and claimed that the exciton response to magnetic fields survives up to the intermediate ferromagnetic phase. This result is of importance. However, as shown in fig. 5a, the spectra shape and width also dramatically changed when the temperature increased. In the present data and analysis, the extracted exciton energy from reflection is not convincing. Adding one of the following data would be helpful:
a) The 2D color map of the reflection spectra, similar to Fig. 2a, which was taken at 120 K or above.
b) Details on extracting the exciton energy from Fig. 5a; error bars are added in Figs. 5 b,c

Version 1:

Reviewer comments:

Reviewer #1

(Remarks to the Author)
I appreciate the authors' efforts to address our previous concerns. I have carefully reviewed the response letter and the revised manuscript. The authors have now taken the spin-canting model with more careful DFT calculations to explain the strong magnetic dependence for the observed resonances in the reflection spectra. They have also performed more polarization dependent data to improve their experimental data quality. Though the manuscript has been improved substantially, I do not feel my concerns have been addressed. Furthermore, their response to the other reviewer does not seem to be sufficient either. Here listed the remained major concerns.
1. While I agree that the new data and theoretical discussion could help confirm the excitonic effect for omega_3 and omega_a, the inter-band transition origins of these excitons remained unclear. Though the authors have mentioned the related discussion of exciton origins in some references and the on-going evolution of the excitonic understanding, the authors still did not explicitly discuss the origins and the transition nature of these excitons. They admitted this excitonic picture is mainly based on previous references, trivializing the impact of this work.
2. Even though the authors have admitted the excitonic picture is based on the previous theoretical references with GW-BSE model, the authors still claimed the magnetic dependence are magneto-excitonic effects, which could be misleading. Without excitonic calculations (such as their specific spatial wavefunction distribution, binding energies, etc), it is not a rigorous way to claim the excitonic effects.
3. I was very curious why the temperature dependence of the delta_E and B_sat is linear. There could be some complex function for this temperature dependence, or the linear dependence may not be a valid approximation. The authors still did not explain how this linear dependence can be obtained from their theoretical model, and continued to use the linear extrapolation for the critical temperature. This is still confusing, and could be very misleading.
4. The other reviewer pointed out the reflection spectra are complex function with many variables (such as dielectric functions, geometry, substrates, etc.), and requested to plot the dielectric spectra. The authors are reluctant to follow this very useful suggestion, making their explanation of their data still questionable. Moreover, there could be more cavity effects for thick CrSBr, which have not been thoroughly discussed in the response letter and the revised manuscript.

Reviewer #2

(Remarks to the Author)
The authors have addressed all concerns. I now recommend the manuscript for publication.

Version 2:

Reviewer comments:

Reviewer #1

(Remarks to the Author)
The authors have addressed all of my suggestions with thorough revisions. I therefore recommend publication.

Open Access This Peer Review File is licensed under a Creative Commons Attribution 4.0 International License, which permits use, sharing, adaptation, distribution and reproduction in any medium or format, as long as you give appropriate credit to the original author(s) and the source, provide a link to the Creative Commons license, and indicate if changes were made.
In cases where reviewers are anonymous, credit should be given to 'Anonymous Referee' and the source.
The images or other third party material in this Peer Review File are included in the article's Creative Commons license, unless indicated otherwise in a credit line to the material. If material is not included in the article's Creative Commons license and your intended use is not permitted by statutory regulation or exceeds the permitted use, you will need to obtain permission directly from the copyright holder.
To view a copy of this license, visit https://creativecommons.org/licenses/by/4.0/

Response to Reviewers

We thank the Reviewers for their time and expertise devoted to our manuscript. Both Reviewers raised several concerns that have helped us significantly improve the work. To address those concerns we have conducted a substantial additional work, both experimental and theoretical. We recognize that the preparation of the revised manuscript has taken considerably longer than typical, but we trust that the extensive scope of additional results we have provided will justify this extended timeline.

The substantially revised version of the manuscript incorporates all of the suggested changes – see the point-by-point response below and the complete fist of changes at the end of this letter. For ease of reading, all Reviewers' original remarks are reprinted in *blue italics*. In view of significant improvements to the revised manuscript that follow from the Reviewers' feedback, we hope that it may now be found suitable for publication in Nature Communications.

Reviewer #1 (Remarks to the Author):

1) The authors have performed high throughput calculations to show the strong spin-orientation dependence of various inter-band transitions (Fig. 4). Though all these transitions show strong magnetic field dependences, the vague assignments of these transitions to the contributions of the observed spectral states are confusing. The qualitative comparisons of calculated inter-band transitions with experimental observations are rough, making the validity of their calculations questionable. The hand-waving explanations with the exciton binding energies is premature. Without careful examination between experiments and calculations, the reasoning of DFT theory is not convincing.

We thank the Reviewer for the constructive feedback. To strengthen our interpretation of the observed transitions we have conducted comprehensive additional studies, both experimental and theoretical, including polarization-resolved optical spectroscopy and supplementary DFT calculations. However, we acknowledge that due to methodological limitations, our primary objective is not to definitively establish the exact origin of these transitions, but rather to identify the main reason for their interesting behavior. Since the emergence of intensive optical studies of CrSBr a few years ago, there has been ongoing scientific discourse regarding the nature of Ω_1, Ω_2, and – more recently – also Ω_3, with the complete picture still evolving. For instance, earlier studies identified the lowest optical excitations (Ω_1, Ω_2) as Wannier excitons with wavefunctions spanning over several nm. [see, e.g., Nature Materials 20, 1657 (2021), Nature 609, 282 (2022), and ACS Nano 17, 5316 (2023)] This spatial delocalization was considered crucial for the interlayer hybridization responsible for their magnetic field dependence (redshift of ~10 meV). However, most recent investigations [e.g. ACS Nano 18, 34235 (2024), arxiv.org/abs/2409.18501 (2024)] suggest a more localized ligand-field origin of those excitations. Similarly, the exact origins of Ω_3 and, particularly, Ω_4 (now Ω_a), have not been fully established. Therefore, we position our work as a contribution to this ongoing scientific discussion, with new experimental and theoretical data, rather than a definitive resolution of the debate. We believe that with our new data, especially regarding Ω_3 and Ω_a transitions, this contribution is a significant step towards understanding the nature of those interesting transitions.

The key findings of our new additional studies are following:
- we have experimentally verified polarization properties of Ω_3 and Ω_a transitions; in particular we have observed that while Ω_3 transition share linear polarization direction with the lowest energy transitions, Ω_a transition is polarized in perpendicular direction;
- our new TDDFT+mBSE calculations (time-dependent density functional theory using the model Bethe–Salpeter equation formalism in the Tamm–Dancoff approximation), which explicitly capture excitonic effects beyond the independent-particle approximation, allowed us to qualitatively

reproduce four transitions that remain if focus of the paper, together with their polarization properties;
- those calculations also allowed us to identify the probable nature of Ω_3 and Ω_a, in particular the momentum states of the band structure responsible for those transitions together with their orbital contributions. In light of those results, the magnitude and the direction of observed Ω_3 and Ω_a shifts are best understood as a direct consequence of the field-driven reconstruction the underlying single-particle band structure.

In the revised version of the manuscript, we have included new experimental results of the polarization-resolved reflectance measurements and replaced the entire calculation-related section with a new theoretical section which elucidates the plausible origin of the large magnetic-field-induced shifts.

2) The phenomenological descriptions of the magnetic field dependences in Equation (1) make the understanding of these origins difficult. Why is field-induced coupling a valid way to describe the magnetic field dependence? Without clarifications on the physical origins, this description is just a mathematical trick.

In describing the magnetic field dependence of the Ω_3 and Ω_4 (now Ω_a) transitions in terms of field-induced coupling, our aim was to qualitatively capture the underlying mechanism. However, given the new findings regarding the origin of these transitions described above, in particular the observation of perpendicular polarization axes of Ω_3 and Ω_a transitions which makes the coupling less likely, we decided to drop such description altogether and instead focus on the conclusions that can be drawn from new experimental data and calculations. In particular, on the fact that the primary reason for the observed substantial red- and blue-shifts of Ω_3 and Ω_a, respectively, is the spin-canting-induced modification of the band structure of the material. Therefore, we have removed the debatable section on the phenomenological descriptions of the magnetic field dependences in Eq. (1) from the revised version of the manuscript.

3) How can the authors make sure that Omega_3 and Omega-4 are excitonic states with large binding energies? Based on the limited experimental data, it can be only inferred these are high-lying excited electronic states. There are no other spectral evidences (like PL, ultrafast dynamics or nonlinear optics) to support the excitonic nature of these two states. The claim of "magneto-exciton effect" is bold, and maybe misleading.

The Reviewer raises an important issue. In fact, our original results did not provide strong evidence for excitonic character of Ω_3 and Ω_4 (now Ω_a). Our tentative assessment was rather based on findings recently published in literature. For example, authors of [ACS Nano 17, 5316 (2023)] identified Ω_1 - Ω_4 (and also higher-energy transitions) as excitonic resonances, based on GW calculations and Bethe–Salpeter equations (BSE). The most recent reports provide further arguments in favor of such interpretation: in [ACS Nano 18, 34235 (2024)] the authors observe weak PL signal from Ω_3 and identify this transition as exciton of the Wannier type (while Ω_1/Ω_2 are recognized as closely related to a Cr^{3+} ligand-field transition, as mentioned above). Similarly, in both [arxiv.org/abs/2409.18437] and [arxiv.org/abs/2409.18501] the authors conclude that Ω_a is an exciton with spatially large wavefunction, basing on PL and/or reflectivity measurements, as well as GW+BSE or more sophisticated, self-consistent QSGW calculations. Our additional calculations included in the revised version of the manuscript are consistent with those findings and further support such picture.

In order to make this issue clear we have modified the manuscript to properly reflect the current state of knowledge presented in the literature and to explain how our results compare to the data already available.

4) The anisotropic axis In Fig. 2d seems to be not along that axis of Fig. 2c. Why is there some angular difference? Does this mean these two states have different anisotropic origins?

In the original manuscript, the small angular difference between the axes in Figs. 2c and 2d fell within our experimental uncertainty. In response to the Reviewer's concerns, we conducted additional measurements using better polarization optics and more precise calibration procedures. These new measurements, now incorporated into the revised Fig. 2, demonstrate nearly perfect alignment of the anisotropic axes.

5) Fig. 4c has very low image resolutions. The label text in these subfigures cannot be resolved.

As a result of our new theoretical studies, we replaced Fig. 4 with new Figs. 5 and 6, and therefore the resolution of Fig. 4 is no longer an issue. We made sure that the resolution of new figures ensures their readability.

6) In Fig. 5b, what does the linear temperature dependence of the energy shifts mean for the physical origins? The authors just showed the linear dependence without any discussion on the physical origins. For Fig. 5b and 5c, extrapolating the linear dependence is taken to infer the ferromagnetic critical temperature. This does not seem to be a valid way, as the temperature-dependent excitonic energy shifts are common and prominent for most semiconductors (even though they are not magnets).

While temperature-dependent excitonic energy shifts are indeed common in most semiconductors, they cannot account for the data presented in Figs. 5b and 5c (now 4b and 4c). There are two key reasons for this:

First, Fig. 4b illustrates the *difference* between the zero-field exciton energy and the energy measured above the saturation magnetic field (B_{sat}). The temperature-induced excitonic energy shift, primarily driven by changes in the energy band gap, should affect both these values similarly in first approximation. Consequently, their difference should remain constant across the investigated temperature range.

Second, temperature-dependent excitonic energy shifts cannot explain the observed temperature dependence of B_{sat}, which is determined exclusively by the magnetic properties of the CrSBr material.

We apologize for any confusion arising from the insufficient explanation in our original manuscript. In the revised version, we have added appropriate comments and clarifications regarding the data presented in Figs 4b and 4c to avoid any such confusion.

Reviewer #2 (Remarks to the Author):

R. Komer studied the reflection spectra of CrSBr under magnetic fields. In the reflection spectra, a peak is significantly red-shifted under fields. This shift is attributed to the colossal magneto-excitonic effects. In addition, DFT simulations were performed to elaborate the experimental spectra. The results are interesting. The paper can be published if the following issues are properly addressed.

We are grateful for the Reviewer's positive assessment of our manuscript and their recognition of our results as interesting. We are particularly encouraged by their recommendation for publication following appropriate revisions.

1) The presented results are reflection contact rather than dielectric function or optical conductivity. This format could lead to misinterpretation of the exciton response. The reflection is governed by the dielectric function of CrSBr, which is determined by the exciton oscillator strength, exciton energy, and damping. All of these parameters could shift the reflection feature. However, the authors attributed the shift solely to exciton energy, without considering other potential mechanisms, such as variation of oscillator strength and change of the exciton line shape. In order to address this concern, which is essential to the key conclusion of this work, the author should extract the optical conductivity or dielectric function. Then, the extracted exciton energies as a function of the magnetic field should be used to illustrate the exciton shift.

The Reviewer correctly points out the importance of the various factors influencing the material's optical response in a magnetic field. To address this point, we have conducted a detailed analysis of the CrSBr dielectric function. Given that extracting the dielectric function from experimental data is considerably more complex than analyzing reflection spectra, we focused our analysis on two specific cases: (i) zero external magnetic field (antiferromagnetic order) and (ii) magnetic field above B_{sat} (ferromagnetic order). In each case we have fitted the experimental reflection spectrum with a calculated one, using the exciton oscillator strength, exciton energy, and damping as fitting parameters. By comparing the results of the analysis of the field-induced excitonic shifts based on the dielectric function and the analysis of the reflection spectra we conclude that the shift of the reflection features observed in the experiment is primarily driven by the excitonic shift and as such can be used – with very good approximation – as a measure of the latter. We have included the discussion of this point in the revised version of the manuscript as well as dielectric function plots in the Supplementary Material of the manuscript.

2) There are multiple peaks in the PL spectra whose origin and correspondence to the reflection spectra are not discussed. They could arise from the Fabry-Perot cavity formed by crystal flakes. The cavity geometry also complicates the reflection spectra, indicating the need to analyze the dielectric function or optical conductivity.

Following the Reviewer's remark, in order to clarify the field dependence of the observed optical transitions, we have analyzed the dielectric function of the studied flakes. The detailed discussion of the dielectric function is now incorporated into the revised version of the manuscript and its Supplementary Material.

3) In fig. 2a, the reflection is enhanced by magnetic fields in the range of 1.9 eV~2 eV. What is the origin of this enhancement? In addition, it seems that the data is overly smoothed., how many spectra are plotted in fig.2a? Raw data may be added to the supplementary materials without any process.

The apparent weak enhancement of the signal above 1.9 eV might arise either due to imperfect normalization of the reflection signal or due to slight modification of the dielectric function of yet unknown origin. Experimentally, the effect is not fully reproducible and for particular combination of the sample and experimental conditions it might even exhibit some asymmetry with respect to the direction of the magnetic field (as seen in Fig. S2 in Supplementary Material), which leads us to believe that the former explanation is more likely. Nevertheless, this issue requires further studies while the main effect that we focus on in our present work is the unusual and strong field-induced shift of the most prominent *sharp* features that is fully reproducible across several samples and robust under variety of experimental conditions.

Regarding Fig. 2a, the figure contains 121 spectra in total, taken at every 0.05T. Since the reflectance spectrum changes very little with such a small field step, the rendered graphics seems smooth even without any additional smoothing algorithm applied. Nevertheless, to avoid any confusion, we have improved the quality of graphics in Fig. 2a. Additionally, together with the manuscript files, we are attaching the raw data uploaded to *figshare* (public data repository partnering with Nature Portfolio).

4) The authors investigated the temperature dependence of spectra and claimed that the exciton response to magnetic fields survives up to the intermediate ferromagnetic phase. This result is of importance. However, as shown in fig. 5a, the spectra shape and width also dramatically changed when the temperature increased. In the present data and analysis, the extracted exciton energy from reflection is not convincing. Adding one of the following data would be helpful:
a) The 2D color map of the reflection spectra, similar to Fig. 2a, which was taken at 120 K or above.
b) Details on extracting the exciton energy from Fig. 5a; error bars are added in Figs. 5 b,c

Following the Reviewer's suggestion, we have included the 2D color map of the reflection spectra taken at elevated temperature in the Supplementary Material of the manuscript. Additionally, we

have also added the error bars in Fig. 5b (now Fig. 4b). Please note that the error bars on Fig 5c (now Fig. 4c) are smaller than the data points used on the plot, as both temperature and the value of the saturation field (B_{SAT}) can be estimated very accurately, regardless of the lower precision of the extraction of the exact value of the transition energy at any given magnetic field.

Complete list of changes in the revised manuscript and Supporting Information:

- We included new experimental results regarding the polarization-resolved reflectance measurements, included in Fig. 2c,d.
- Following our new theoretical results, we replaced the previous section "*DFT Calculation of the Band Structure,"* which relied, among other things, on qualitative estimates of exciton binding energies, with a new section entitled "*Theoretical perspective on magnetic-field–induced higher-band exciton shifts,"* which elucidates the plausible origin of the large magnetic-field-induced shifts.
- We replaced Figure 4 (*Electronic band structures obtained within the DFT+U+SOC approach*) with new Figures 5 and 6, which illustrate the orbital composition of these excitonic states and the evolution of the electronic structure mimicking the applied magnetic field, respectively.
- We included new results of a detailed analysis of the CrSBr dielectric function in the Supplementary Metarial and suitable comments in the revised version of the manuscript.
- We removed the debatable section on the phenomenological description of the magnetic field dependences in Eq. (1) and in exchange we expanded the conclusions that can be drawn from new experimental data and calculations – namely the discussion on the primary reason for the observed substantial red- and blue-shifts of Ω_3 and Ω_a being the spin-canting-induced modification of the band structure of the material.
- We clarified the discussion of the excitonic character of the observed transitions to properly reflect the current state of knowledge presented in the literature and to explain how our results compare to the data already available.
- We added additional comments and clarifications regarding the data presented in Figs. 5b and 5c (now 4b and 4c, respectively), related to the role of the temperature-induced changes in the energy band gap in the observed energy shifts.
- We have included the 2D color map of the reflection spectra taken at elevated temperature in the Supplementary Material.
- We have added the error bars in Fig. 5b (now Fig. 4b).
- We expanded the *Computational Details* section in the Supporting Information by adding a new subsection "*Excitonic calculations (TDDFT+mBSE)."* In this part, we describe our approach in detail, dividing it into: *Ground-State Calculations*, *Double-Screened Hybrid (DSH) Functional*, and *Excitonic and Optical Calculations.*
- We added a new Figure S6, which illustrates the excitonic wavefunctions corresponding to the first bright excitonic states (from left to right: $\Omega_1/_2$, Ω_3, and Ω_a), projected onto the band states. We also present orbital-resolved band projections for Cr, S, and Br atomic states for both A-AFM and FM phases. Additionally, we included a new Figure S7 showing the imaginary part of the dielectric function for two magnetic orderings of bulk CrSBr.
- We added two new co-authors, Mr. Mateusz Raczyński and Mr. Karol Gałązka, as they contributed to additional experiments and calculations, respectively. Mr. Raczyński contributed to the analysis of the CrSBr dielectric function, while Mr Gałązka contributed to the analysis and post-processing of the excitonic calculation results.

We are grateful to both Reviewers for the time and expertise they dedicated to evaluating our work. We are pleased to learn that Reviewer #2 is satisfied with our responses to their previous remarks and the revisions introduced to the manuscript, and considers all prior concerns adequately addressed. We equally appreciate the constructive comments and suggestions provided by Reviewer #1, which we believe have contributed to a further strengthening of the manuscript.

The revised version of the manuscript addresses all outstanding remarks in full. A point-by-point response is provided below, followed by a comprehensive list of changes at the end of this letter. For ease of reference, each Reviewer's original comment is reprinted in *blue italics*.

In light of the reports from both Reviewers and the resulting changes incorporated into the revised manuscript, we hope that it may now be considered suitable for publication in Nature Communications.

Reviewer #1 (Remarks to the Author):

1. While I agree that the new data and theoretical discussion could help confirm the excitonic effect for omega_3 and omega_a, the inter-band transition origins of these excitons remained unclear. Though the authors have mentioned the related discussion of exciton origins in some references and the on-going evolution of the excitonic understanding, the authors still did not explicitly discuss the origins and the transition nature of these excitons. They admitted this excitonic picture is mainly based on previous references, trivializing the impact of this work.

While we believe that our experimental and theoretical data contribute to the ongoing scientific discussion regarding the origin of the higher-energy optical transitions in CrSBr, the central observation of our work is the peculiar behavior of Ω_3 and, particularly, Ω_a in magnetic field. Although the exact origin of those transitions has not been fully established, all available data and calculations point to their excitonic nature (see, for example, references 18, 19, and very recent 22, 23 published during the review process of our work). Our data, in particular our TDDFT+mBSE calculations (resulting in the decomposition of the Ω_3 and Ω_a wavefunctions in the band basis together with their orbital contributions (Figs. 5 and S7)), fully agree with that picture. Nevertheless, the most important and impactful part of our work is the observation of particularly strong shift of those transitions in magnetic field, opposite for Ω_3 and Ω_a, combined with their opposite linear polarizations, and discussion of possible origins of that observations. In light of our DFT+U+SOC calculations we conclude that the magnitude and the direction of observed Ω_3 and Ω_a shifts are best understood as a direct consequence of the field-driven reconstruction of the underlying single-particle band structure – a mechanism distinct from that primarily responsible for previously reported field-dependent redshift of fundamental excitonic transitions.

We hope that those new results will spark discussion on the unusual behavior of the Ω_3 and Ω_a and fuel future, more refined theoretical work that, for example, fully accounts for the spin-orbit coupling beyond the independent-particle approximation. We have added suitable comments to the manuscript to emphasize such point of view.

2. Even though the authors have admitted the excitonic picture is based on the previous theoretical references with GW-BSE model, the authors still claimed the magnetic dependence are magneto-excitonic effects, which could be misleading. Without excitonic calculations (such as their specific spatial wavefunction distribution, binding energies, etc), it is not a rigorous way to claim the excitonic effects.

We believe that the core issue here is the use of the term "magneto-excitonic" for the description of the effects we observe. While we have adapted such term from prior publications on CrSBr (e.g. J. Shi et al., ACS Nano 19, 29977 (2025)), we now recognize that such naming might be misleading depending on the reader background. In particular, the transitions we observe have different nature from

magneto-excitons traditionally studied in the high-magnetic field regime, particularly in low-dimensional systems, when a clear Landau level quantization is observed and the distance between the Landau levels greatly exceeds the binding energy of a zero-field exciton (e.g. A. V. Korolev and M. A. Liberman, Int. J. Mod. Phys. B 10, 729 (1996)). In our case, Landau level quantization is not visible since the cyclotron frequency at ~2T is too small, and the optical transitions we observe cannot be considered as transitions between distinct Landau levels. We thank the Reviewer for bringing up this issue. In order to avoid confusion and potentially misleading expressions we modified the language throughout the manuscript (including the title of the manuscript) and replaced the term "magneto-excitonic effects" with more suitable "magneto-optical effects", or similar.

3. I was very curious why the temperature dependence of the delta_E and B_sat is linear. There could be some complex function for this temperature dependence, or the linear dependence may not be a valid approximation. The authors still did not explain how this linear dependence can be obtained from their theoretical model, and continued to use the linear extrapolation for the critical temperature. This is still confusing, and could be very misleading.

While our model does not account for the temperature-related effects, the linear temperature dependence of ΔE for fundamental optical transitions in CrSBr has been discussed in Ref. 16. In that paper the authors identify such temperature dependence as determined by the population of incoherent magnons and extending well beyond the Neel temperature of the material – up to T_{corr}≈180K (which is the temperature up to which magnetic field correlations are observed). Therefore, in our work we now only use the temperature dependence of ΔE of Ω_3 as yet another confirmation of connection between that transition's field dependence and the magnetic order within the crystal lattice, as both the linear dependence and the critical temperature (~185K in our case) are nearly identical as those found for fundamental transition. We added suitable comments to the manuscript to clarify this issue.

4. The other reviewer pointed out the reflection spectra are complex function with many variables (such as dielectric functions, geometry, substrates, etc.), and requested to plot the dielectric spectra. The authors are reluctant to follow this very useful suggestion, making their explanation of their data still questionable. Moreover, there could be more cavity effects for thick CrSBr, which have not been thoroughly discussed in the response letter and the revised manuscript.

In response to Reviewer #2's initial request, we included plots of the imaginary part of the dielectric function (ε_2) in the Supplementary Material (Fig. S1), accompanied by an appropriate comment in the main text. Although Reviewer #2 has confirmed that all their concerns have been fully addressed, we have taken the opportunity to further clarify the dielectric spectra issue by also adding the real part of the dielectric function (ε_1) to Fig. S1, which now presents complete information on this issue. Regarding cavity effects, although they may influence the reflectance spectrum near the exciton resonances, and in fact we do observe the characteristic additional dips in the vicinity of the first higher-energy transition that could likely be identified as distinct polaritonic branches in thick CrSBr flake, those features shift commensurately with the main peak (similarly to the case of the rich spectrum of the fundamental excitons at 1.3-1.4 eV) and as such do not affect our analysis of the *relative* field-induced shifts. We have added this comment to the manuscript to clarify this issue.

Reviewer #2 (Remarks to the Author):

The authors have addressed all concerns. I now recommend the manuscript for publication.

We thank the Reviewer for re-evaluating the revised manuscript and for their positive assessment. We are pleased to learn that all previous concerns have been properly addressed and that the Reviewer recommends the manuscript for publication in Nature Communications.

Full list of changes made in the updated version of the manuscript and Supporting Information:

- We have revised the language throughout the manuscript (including the title) to eliminate potentially misleading expressions such as "magneto-excitonic effects".
- We have expanded the discussion of our theoretical results in the context of the excitonic nature of the higher-energy transitions, and clarified that the central result of our work is the observation of peculiar behavior of Ω_3 and, particularly, Ω_a.
- We have added comments on the linear temperature dependence of ΔE and the critical temperature in Fig. 4, and elaborated on the similarities between Ω_3 and fundamental excitonic transitions in that context.
- We have included a remark on the cavity effects and their influence on the observed reflectance spectra.
- We have expanded Fig. S1 with new data to provide a complete account of the dielectric function of CrSBr.
- We have added three new references to most recent publications on CrSBr to reflect the current state of knowledge.
- We have made several minor corrections to the text and figures to remove previously unnoticed errors and improve overall readability.
